# Bladder Cancer Biomarker Discovery Using Global Metabolomic Profiling of Urine

**DOI:** 10.1371/journal.pone.0115870

**Published:** 2014-12-26

**Authors:** Bryan M. Wittmann, Steven M. Stirdivant, Matthew W. Mitchell, Jacob E. Wulff, Jonathan E. McDunn, Zhen Li, Aphrihl Dennis-Barrie, Bruce P. Neri, Michael V. Milburn, Yair Lotan, Robert L. Wolfert

**Affiliations:** 1 Clinical Research and Development, Metabolon Inc., Durham, North Carolina, United States of America; 2 Department of Urology, University of Texas Southwestern Medical Center, Dallas, Texas, United States of America; Johns Hopkins University, United States of America

## Abstract

Bladder cancer (BCa) is a common malignancy worldwide and has a high probability of recurrence after initial diagnosis and treatment. As a result, recurrent surveillance, primarily involving repeated cystoscopies, is a critical component of post diagnosis patient management. Since cystoscopy is invasive, expensive and a possible deterrent to patient compliance with regular follow-up screening, new non-invasive technologies to aid in the detection of recurrent and/or primary bladder cancer are strongly needed. In this study, mass spectrometry based metabolomics was employed to identify biochemical signatures in human urine that differentiate bladder cancer from non-cancer controls. Over 1000 distinct compounds were measured including 587 named compounds of known chemical identity. Initial biomarker identification was conducted using a 332 subject sample set of retrospective urine samples (cohort 1), which included 66 BCa positive samples. A set of 25 candidate biomarkers was selected based on statistical significance, fold difference and metabolic pathway coverage. The 25 candidate biomarkers were tested against an independent urine sample set (cohort 2) using random forest analysis, with palmitoyl sphingomyelin, lactate, adenosine and succinate providing the strongest predictive power for differentiating cohort 2 cancer from non-cancer urines. Cohort 2 metabolite profiling revealed additional metabolites, including arachidonate, that were higher in cohort 2 cancer vs. non-cancer controls, but were below quantitation limits in the cohort 1 profiling. Metabolites related to lipid metabolism may be especially interesting biomarkers. The results suggest that urine metabolites may provide a much needed non-invasive adjunct diagnostic to cystoscopy for detection of bladder cancer and recurrent disease management.

## Introduction

In the U.S., bladder cancer is the 4^th^ most common cancer type in men and the 11^th^ most common cancer type in women [1]. In the U.S. for 2012, it was estimated that 73,000 new cases would be diagnosed and 15,000 people would die from the disease [1]. Patients with bladder cancer most frequently present with hematuria [2]. Diagnosis of bladder cancer, in those patients presenting with hematuria, primarily involves cystoscopy along with imaging, cytology and biopsy [3]. Cystoscopy and cytology are the current standards for initial diagnosis and recurrence, but limitations exist. Cystoscopy may fail to visualize certain areas within the bladder and may also fail to detect all cancers, particularly some cases of carcinoma in situ [4]. Cytology has high specificity and selectivity for high grade tumors but fails to provide strong predictive value for low grade tumors [5]. Treatment options are based on staging and whether there is muscle tissue invasion. A majority of bladder cancers (75%) are urothelial carcinomas classified as non-muscle invasive bladder cancers (NMIBC). In NMIBC, approximately 70% of patients present with stage pTa, 20% with pT1 and 10% with carcinoma in situ (CIS) [6]. The recurrence rate for NMIBC after tumor resection is high, with estimates ranging from 35 to 80% [6], [7]. Due to risk of tumor recurrence or progression, established guidelines recommend that NMIBC patients be monitored after initial diagnosis and treatment [8], [9]. A regular schedule of cystoscopy is recommended for surveillance at a frequency of every 3–6 months for 3 years and yearly there after [10], [11]. As a result, bladder cancer can be viewed as a chronic disease with life-long follow-up required. Long term surveillance relying on cystoscopy, besides being invasive, has the potential for adverse events and can involve considerable long term expenses [12], [13]. In addition, patient aversion to cystoscopy may result in reduced patient compliance with regular surveillance recommendations [14]. There is a strong clinical need for a non-invasive, inexpensive alternative to cystoscopy which will aid in the detection of primary cancers, monitor recurrence and help stratify patients as to risk of recurrence and progression. Recent advances in metabolomics have opened up the possibility of using urine metabolites as biomarkers for cancer [15]–[18]. A number of studies have compared metabolite differences in bladder tumors relative to benign tissue and have identified candidate cancer biomarkers [19]–[23]. One study also examined differences in urine metabolites between patients presenting with bladder cancer relative to cancer free controls [19]. Earlier studies were often limited in the number of detected named metabolites and a more comprehensive metabolite profiling may yield new candidate biomarkers and predictive algorithms. We report here the metabolomic profiling of urine from two cohorts of bladder cancer patients and their respective non-cancer controls. The data suggest multiple candidate bladder cancer biomarkers which may offer prognostic value in identifying cancer positive urines.

## Materials and Methods

### Patient Selection

Retrospective (cohort 1) and prospective (cohort 2) urine sample sets were obtained from an IRB-approved urine repository (IRB #CR00008160/STU032011-187) at the University of Texas Southwestern Medical Center (UTSW). All subjects were consented with written consents. Cohort 1 bladder cancer positive urines were from subjects presenting with either primary or recurring cancer. Voided urine samples were obtained prior to cystoscopy for subjects from cohort 1 bladder cancer positives, along with cancer history and hematuria controls. Cystoscopies were conducted as part of ongoing surveillance or for cancer detection and results were used to diagnose current cancer status, either present or absent. Cohort 2 urine samples were obtained from subjects presenting with hematuria or from subjects with a history of disease undergoing surveillance. Bladder cancer positive urines in cohort 2 were obtained from subjects presenting with either primary or recurrent disease. Metadata regarding age, gender, race, and cancer stage and grade was available for both cohorts.

### Metabolomic Profiling

The mass spectrometer platforms, sample extraction and preparation, instrument settings and conditions, and data handling have been previously described in detail [24]. Briefly, the major components of the process can be summarized as follows. Osmolality of each urine sample is determined prior to processing. A cocktail of recovery standards was added to the urine samples and 100 uL aliquots were extracted in 500 uL methanol. The resulting extract was divided into three fractions for untargeted metabolic profiling and randomized for analysis. Each sample was dried under vacuum to remove organic solvent. Samples were characterized using three independent platforms: ultrahigh-performance liquid chromatography/tandem mass spectrometry (UHPLC-MS/MS) in the negative ion mode, UHPLC-MS/MS in the positive ion mode and gas chromatography-mass spectrometry (GC-MS) after sialylation. The reproducibility of the extraction protocol was assessed by the recovery of the xenobiotic compounds spiked in every urine sample prior to extraction. Cohort 1 urines were analyzed using a platform consisting of a Waters ACQUITY UHPLC (Waters Corporation, Milford, MA, USA) and a Thermo-Finnigan LTQ mass spectrometer (Thermo Fisher Scientific Inc., Waltham, MA. USA), while cohort 2 was analyzed using a platform consisting of a Waters ACQUITY UHPLC and a ThermoFisher Scientific Orbitrap Elite high resolution/accurate-mass mass spectrometer (Thermo Fisher Scientific Inc., Waltham, MA. USA). Compounds were identified by comparison to library entries of purified standards or recurrent unknown entities. Identification of known chemical entities was based on comparison to metabolomic library entries of purified standards based on chromatographic properties and mass spectra. As of this writing, more than 4000 commercially available purified standard compounds had been acquired and registered into the LIMS for distribution to both the LC and GC platforms for determination of their analytical characteristics. Additional entities (unnamed compounds) were identified by virtue of their recurrent nature (both chromatographic and mass spectral). These compounds have the potential to be identified by future acquisition of a matching purified standard or by classical structural analysis.

### Statistical analysis

All statistical analyses were performed in R version 2.14.2 [25]. Wilcoxon Test was used to determine the statistical significance of metabolite mean differences between comparator groups. For all analyses, missing values (if any) were imputed with the observed minimum for that particular compound (imputed values were added after block-normalization). The statistical analyses were performed on natural log-transformed data to reduce the effect of any potential outliers in the data. In addition, data was normalized to sample osmolality to compensate for differences in urine concentration. Random forest is a supervised classification technique based on an ensemble of decision trees [26] and was performed in R version 2.14.2. Hierarchical clustering of bladder cancer and control urine abundance profiles was performed in ArrayStudio version 5.0 using complete linkage and Pearsons's correlation as the similarity metric (OmicSoft, Raleigh, NC). Calculations of AUCs and ROC curves were performed using the pROC package in R [27]. The multi-biochemical algorithm, used to generate AUCs and ROC curves, was trained and tested from data that was rescaled so that the medians of both the cohort-1 and cohort-2 negatives were equal to 1. The rescaling permits algorithm testing on a scale appropriate for the fitted coefficients derived from the training set.

## Results

### Subject populations

Urine metabolic profiling was performed on two subject cohorts. Cohort 1 was utilized as an exploratory/biomarker identification set to identify biochemicals whose levels were different in the urines of bladder cancer urines relative to levels in control urines. Cohort 2 was utilized as a second discovery set and to test the predictive value of candidate biomarkers selected from the cohort 1 data set, but since cohort 2 samples were analyzed on a more sensitive mass spec platform, metabolites that were only measured in the cohort 2 samples were also of interest. Cohort 1 was a retrospective urine sample set collected at the University of Texas Southwestern, while cohort 2 samples were collected prospectively at the same institution. A summary of patient demographics for the two cohorts is presented in Table 1. Cohort 1 comprised 66 urines from subjects diagnosed with BCa and 266 non-BCa controls. Urines in cohort 1 were collected from subjects with either primary or recurrent disease. Some differences in overall gender and race compositions were present in cohort 1. Non-BCa controls in cohort 1 can be subdivided into three populations: 1) subjects presenting with hematuria; 2) subjects with a history of BCa, but no current disease and 3) normal subjects with no history of BCa. Cohort 2 was comprised of 29 urines from subjects diagnosed with BCa and 79 non-BCa controls. As in cohort 1, there were some differences in gender and racial balance between the BCa and non-BCa controls. Cohort 2 urines were obtained from subjects with either primary or recurrent disease in a ratio identical to that of cohort 1 (59% recurrent: 41% primary). Also of note was a cohort difference in the percent of high grade vs. low grade BCa tumors, with cohort 1 having a much higher percentage of high grade BCa (79%) than cohort 2 (59%).

**Table 1:** Cohort Subject Demographics.

	Cohort 1		Cohort 2	
	BCa	Non-BCa	BCa	Non-BCa
**# of subjects**	66	266	29	79
**Age**	67.4	64.2	66.7	65.1
**Gender**				
Male	56 (85%)	169 (64%)	23 (79%)	43 (54%)
Female	9 (14%)	96 (36%)	6 (21%)	36 (46%)
Unknown	1	1		
**Race**				
White	53 (80%)	180 (68%)	27 (94%)	62 (79%)
Black	4 (6%)	43 (16%)		11 (14%)
Asian	2 (3%)	12 (5%)		1 (1%)
Hispanic	6 (9%)	24 (9%)	1 (3%)	4 (5%)
Unknown	1 (2%)	7 (3%)	1 (3%)	1 (1%)
**BCa Grade**				
High	52 (79%)		17 (59%)	
Low	4 (6%)		10 (34%)	
Not available	10 (15%)		2 (7%)	
**BCa Stage**				
Ta	5 (8%)		15 (52%)	
Tis	5 (8%)			
T1	11 (17%)		5 (17%)	
T2	13 (20%)		4 (14%)	
T3	22 (33%)			
T4	8 (12%)		2 (7%)	
Unknown	2 (3%)		3 (10%)	
**BCa Recurrent**	39 (59%)		17 (59%)	
**BCa Primary**	27 (41%)		12 (41%)	
**Non-BCa controls**				
Hematuria		58 (22%)		20 (25%)
History - no current		119 (45%)		59 (75%)
Normal		89 (33%)		

### Metabolomic profiling and analysis

Urine samples were extracted and metabolic profiling was performed using positive (+) and negative (−) LC-MS/MS and also GC-MS, to obtain broad coverage of the biochemicals present. MS peaks were identified using Metabolon's proprietary peak integration/identification software, by comparing MS peak data to that of a library of purified standards or recurrent unknown entities. Following imputation of minimum observed values, log transformation and normalization procedures, statistical analysis was performed to identify statistically significant differences in metabolite levels between comparator groups. Profiling of cohort 1 measured 499 named and 624 unnamed biochemicals, while profiling of cohort 2 measured 587 named and 541 unnamed biochemicals. Lists of all named metabolites measured in the two cohorts are shown in S1 & S2 Tables. The increased number of named compounds measured in cohort 2, relative to cohort 1, in part, reflects the greater sensitivity of the accurate mass MS instrument used for cohort 2 and an expansion of the biochemical library in the time period between profiling cohorts 1 and 2. A Wilcoxon two sample test was employed to identify statistically significant differences in metabolite levels in cohort 1 BCa urines relative to control urines. Statistical analysis was performed comparing BCa urines to all control groups combined; or comparing BCa to each of the control subgroups. The number of statistically significant differences in named compound levels ranged from 178 to 233 across the different comparisons (Table 2). Overall, the number of statistically significant biochemicals did not vary greatly when comparing BCa positive samples to the different control groups. Analysis of cohort 2 BCa urines vs. controls, using a Wilcoxon test, identified 75 named biochemicals as displaying statistically significant differences with 70 biochemicals elevated and 5 biochemicals lower in BCa urines relative to control urines (Table 2). The smaller number of statistically significant differences in cohort 2 relative to cohort 1 may reflect, in part, the lower sample numbers in cohort 2. The higher percentage of higher stage tumors in cohort 1 relative to cohort 2 may have also impacted the number of statistically significant differences observed.

**Table 2:** Statistically significant differences in metabolite levels between comparator groups.

Wilcoxon Test	Cohort 1				Cohort 2
	BCa/Normal	BCa/History	BCa/Hemat	BCa/All Cnt	BCA/All Cnt
Total biochemicals P≤0.05	570	574	488	616	107
Biochemicals (↑|↓)	128|442	139|435	96|392	157|554	87|20
Total named biochemicals p≤0.05	233	216	178	231	75
Named biochemicals (↑|↓)	71|162	67|149	45|133	65|166	70|5

Cohort 1 All Cnt (all controls) represents combined data for BCa negative: normal, history and hematuria samples. Cohort 2 All Cnt group represents combined data for BCa history and hematuria samples.

### Identification of candidate biomarkers

**Figure 1:**
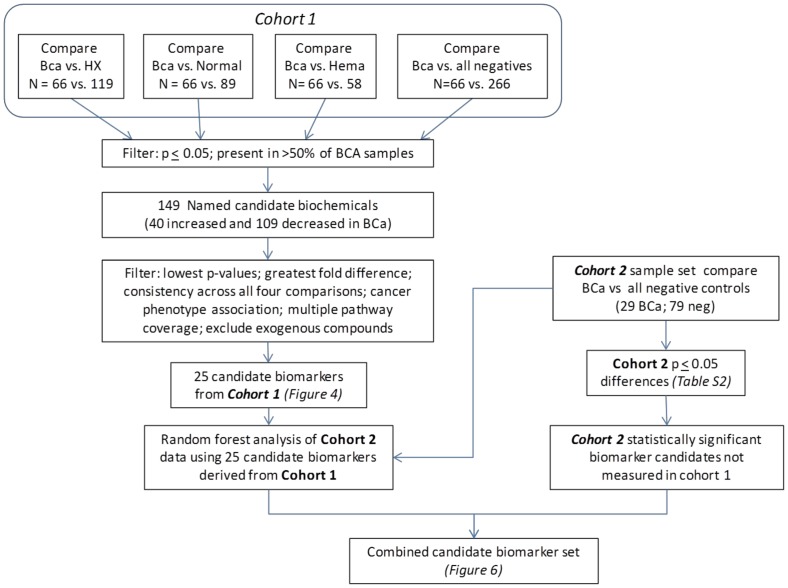
Workflow diagram of biomarker candidate selection from both cohort 1 and cohort 2 data sets. Abbreviations: HX, BCa negative but with history of BCa; Hema, BCa negative presenting with hematuria.

**Figure 2:**
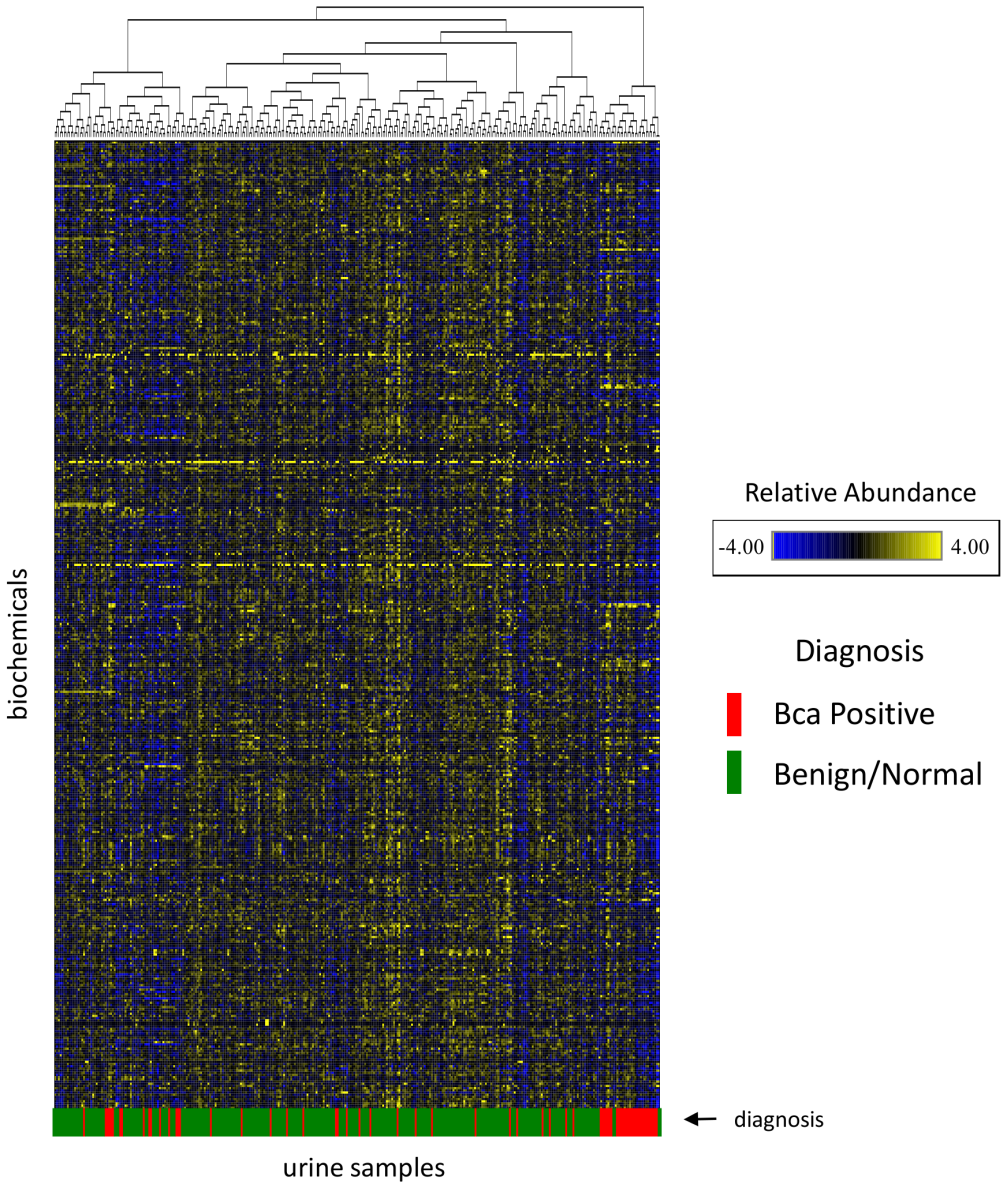
Hierarchical clustering of cohort 1 samples (N-332) and all named biochemicals exclusive of drugs (N-422). Subject BCa diagnosis (post urine collection) is indicated in the lower bar. Clustering was performed using complete linkage and Pearsons's correlation as the similarity metric.

A strategy was employed to use cohort 1 to identify candidate biomarkers and to rank the most interesting cohort 1 biomarkers for BCa predictability using the cohort 2 samples set. A workflow diagram of the strategy used for biomarker testing and confirmation is displayed in Fig. 1. Hierarchical clustering was performed on the cohort 1 data set using all samples (332) and all named biochemicals, excluding exogenous drugs (total  = 442). The results of the hierarchical clustering are presented in Fig. 2, with some degree of BCa sample clustering observed. The clustering results suggest that metabolite differences between the cancer and no-cancer groups exist and that these differences have a capacity to differentiate the urine samples.

### Selection of biomarker candidates from the cohort 1 data set

**Figure 3:**
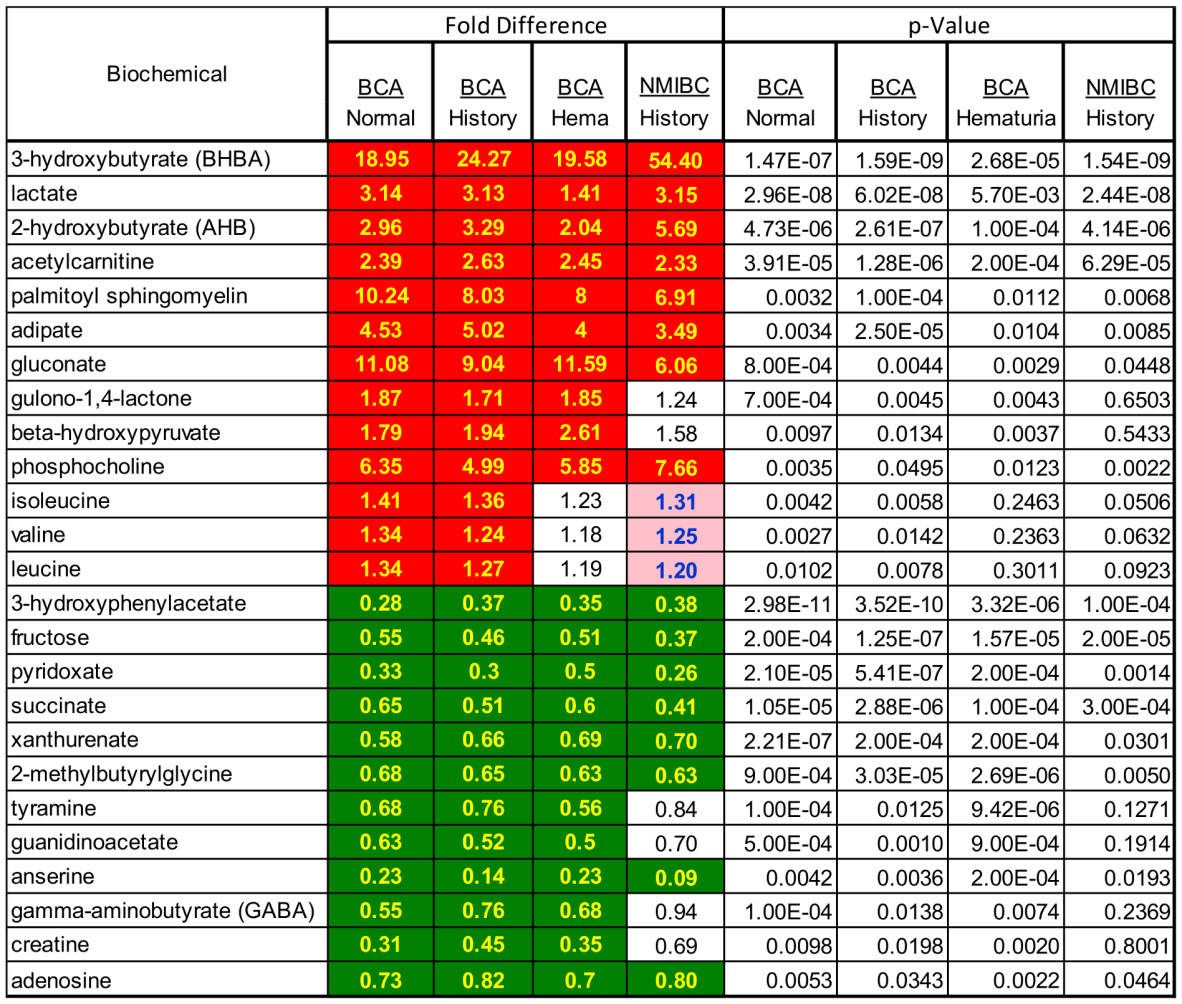
Cohort 1 derived candidate biomarker set heatmap for BCa vs. control groups. Red fill cells indicate metabolites with higher mean levels in BCA urines than in non-BCa controls at a p≤0.05 significance. Green cells indicate lower levels in BCa relative to control urines at a p≤0.05 significance. Statistical q-values and profiling results for all other named compounds measured in cohort 1 samples are presented inn S1 Table.

**Figure 4:**
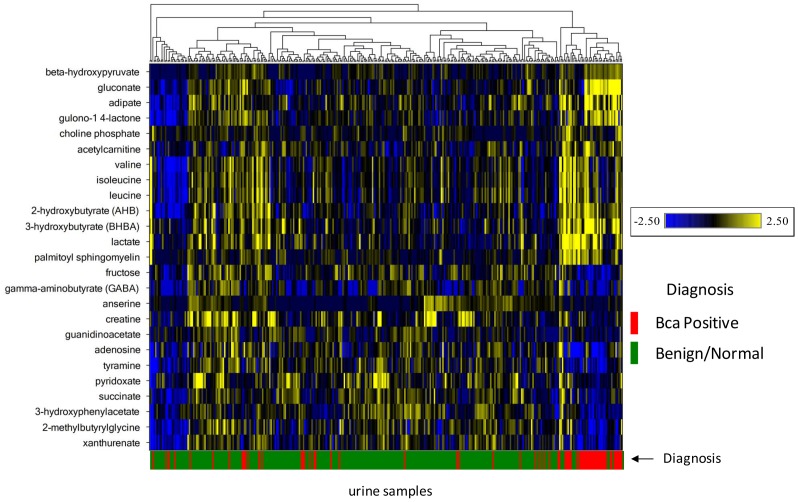
Hierarchical clustering of cohort 1 samples (N = 332) and the set of 25 candidate biomarkers. Subject BCa diagnosis (post urine collection) is indicated in the lower bar. Clustering was performed using complete linkage and Pearsons's correlation as the similarity metric.

A Wilcoxon test was applied to cohort 1 profiling data comparing urines from subjects with current BCa to urines from three separate control groups: 1) subjects presenting with hematuria; 2) subjects with a history of BCa but no current disease or 3) normal subjects with no history of BCa. In addition, a Wilcoxon test was applied comparing BCa urines to a control group consisting of all non-BCa urines combined. A heatmap and statistics for all measured metabolites for the 4 different comparisons is contained in S1 Table. Combined, 290 statistically significant differences in named metabolite levels were identified between the 3 separate BCa/comparator analyses, with 135 metabolites displaying statistically significant differences across all three BCa to negative control comparisons (hematuria, history, normal; S1 Table). To reduce the total number of metabolite differences down to a more manageable set of “best biomarker candidates”, several filtering criteria were applied. Filters included: 1) metabolites with BCa to control levels that were statistically significant in at least 3 of 4 BCa to control group comparisons; 2) metabolite differences displaying the lowest p-value (all p≤0.05); 3) greatest fold differences between BCa and controls; 4) measured in >50% of urine samples; 5) cancer phenotype association; 6) coverage of multiple metabolic pathways; 7) named compounds only; 8) exclusion of exogenous compounds (e.g. xenobiotics, drugs). Applying these selection criteria we designated a panel of 25 candidate biomarkers for further analysis. The set of 25 candidate biomarkers is shown in the heatmap of Fig. 3, along with the statistical performance in each of 3 possible BCa to control group comparisons. Also shown in Fig. 3 is a bladder cancer subset analysis comparing only non-muscle invasive bladder cancers to the history control group. In comparing all BCa samples to each of the control groups, all biochemicals with the exception of the branched-chain amino acids (BCAA) leucine, isoleucine and valine displayed p≤0.05 statistical significance in all 3 control group comparisons. 3-hydroxybutyrate and gluconate were the most highly elevated in BCa urines, while anserine and 3-hydroxyphenylacetate and pyridoxate were most reduced in BCa vs. control urines. The majority of biomarker candidates which achieved statistical significance when all BCa samples were compared to the history controls also displayed statistically significant differences when only NMBIC samples were compared to the history controls. Differentiation of NMBIC cancers is important because they will be more prevalent in patients under active surveillance. The 25 candidate biomarkers selected from the cohort 1 data were used in a hierarchical clustering analysis of cohort 1 samples. Clustering of BCa and control samples was observed, indicating that differential levels of the 25 biochemicals offer some degree of urine sample stratification based on diagnosis (Fig. 4).

### Cohort 1 candidate biomarkers that best differentiate cohort 2 samples

**Figure 5:**
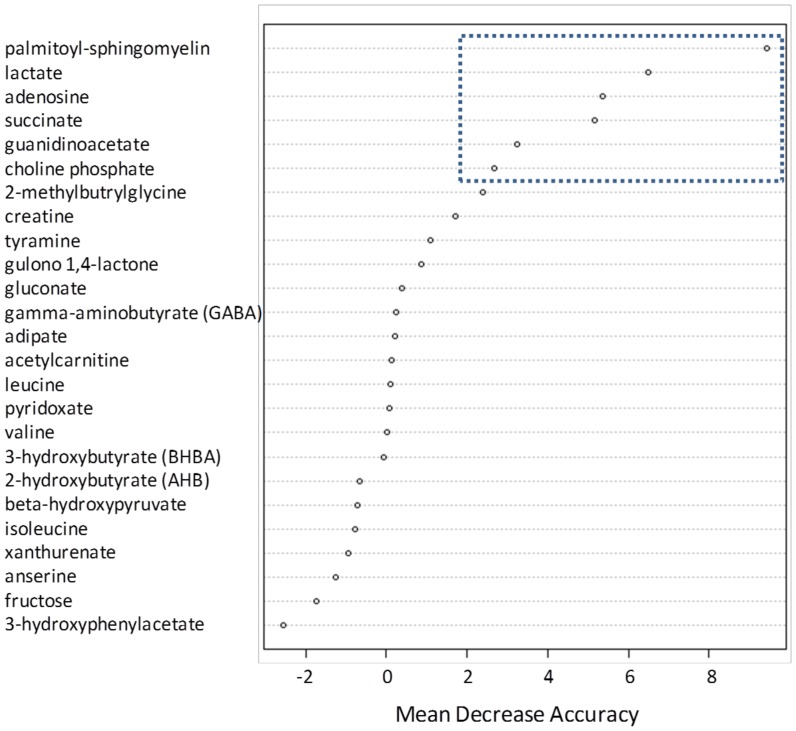
Random Forest analysis of cohort 2 sample data using 25 metabolites selected from cohort. Metabolites are rank-ordered by their mean decrease accuracy score. A higher mean decrease accuracy value indicates a greater predictive value. The 6 boxed data points represent top performing metabolites summarized in Fig. 6.

**Figure 6:**
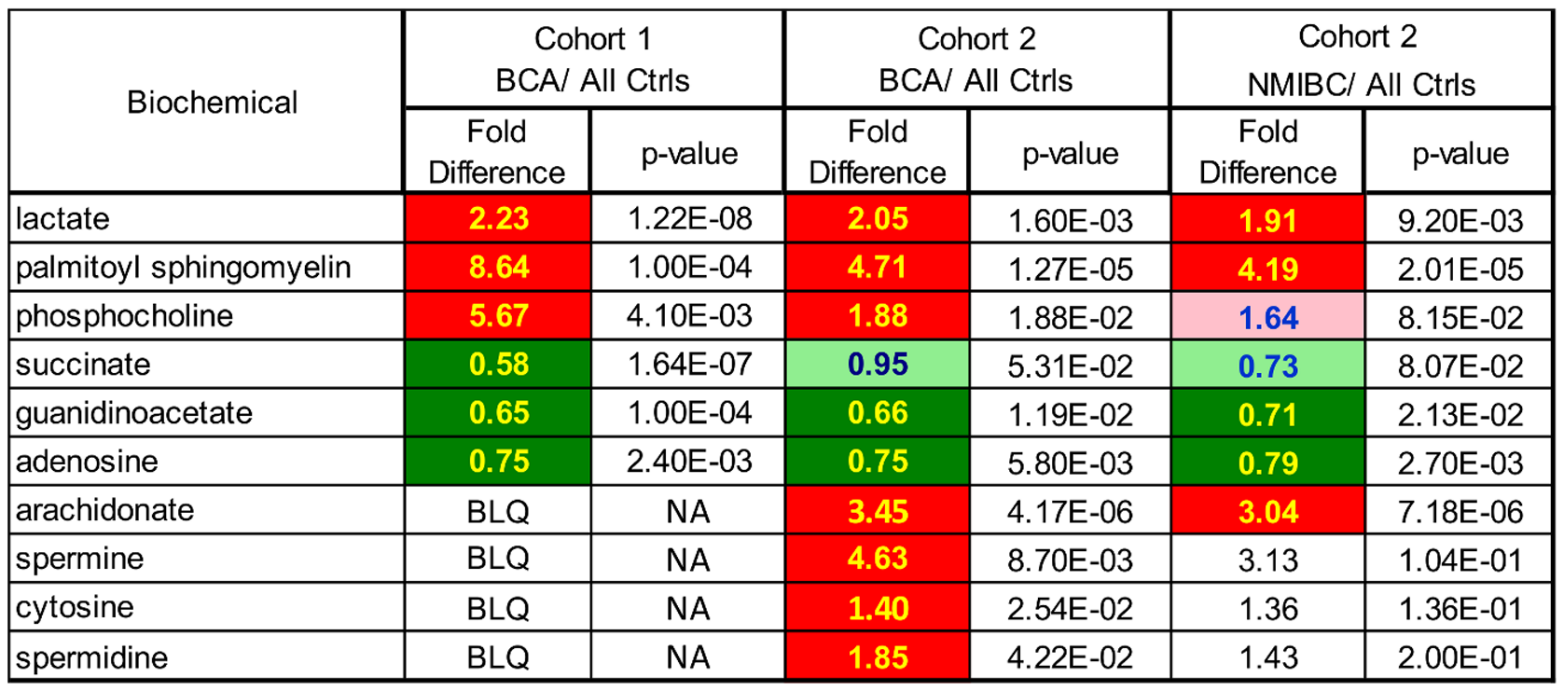
Comparison of statistically significant metabolites from cohorts 1 and 2. Comparisons are for all BCA positive urines versus combine BCA negative controls. Dark red and dark green cells represent fold differences with a p≤0.05. Light green cell with blue text represents p≤0.1. BLQ: below limit of quantitation; NA: not applicable.

A random forest analysis was conducted using the 25 cohort 1 biomarker candidates to stratify the cohort 2 sample set into their proper cancer and non-cancer groups. Random forest is an ensemble method based on classification trees and the out-of-bag error gives an estimate of how well we can expect to predict a future sample. The random forest analysis provides an “importance” rank ordering of biochemicals. The relative importance of each of the 25 metabolites is shown in Fig. 5, with palmitoyl sphingomyelin displaying the greatest discriminatory power (higher mean decrease accuracy value). The top 6 discriminatory metabolites in the random forest analysis constituted 3 metabolites which were higher in BCa urines and 3 that were lower in BCa samples. A comparison of relative levels for these 6 metabolites in all cancer urines versus all non-cancer controls in the two cohorts is displayed in Fig. 6. The differences in relative levels for each of the 6 metabolites was statistically significant (p≤0.05) in both cohorts, with the exception of succinate which achieved a p-value of 0.053 in the cohort 2 comparison. In addition, the NMIBC subset of BCA samples was compared to all control samples and 4 of the 6 metabolites continued to achieve statistical significance, at a p≤0.05 level, with phosphocholine and succinate being the exception (Fig. 6). Phosphocholine and succinate were statistically significant at a p≤0.1 level.

### Additional biomarker candidates observed in cohort 2 urine samples

Metabolic profiling of cohort 2 urine samples was performed using a more sensitive accurate- mass MS-platform, which is capable of measuring urine metabolites present at lower concentrations. A heatmap containing all named metabolites measured in cohort 2 samples is presented in S2 Table. Arachidonate, spermidine, spermine and cytosine, were not measured in cohort 1 urines, but were elevated in cohort 2 BCa urines at p≤0.05 (Fig. 6). Arachidonate was also elevated in NMIBC tumor urines to a statistically significant level, when the NMIBC samples were segregated and analyzed separately from MIBC tumor urines (Fig. 6). Spermine, spermidine and cytosine were elevated in NMIBC urines as well, but not at statistically significant levels. These four metabolites may also be considered to be candidate biomarkers, but confirmation would require an independent cohort that had also been profiled on the accurate-mass instrument.

### Multi-analyte algorithm performance using a set of 6 biomarkers

**Figure 7:**
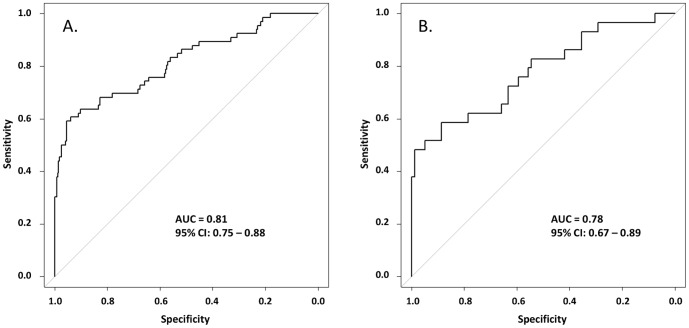
Receiver Operating Characteristic curves for a 6-biomarker algorithm. An algorithm, utilizing the candidate biomarkers palmitoyl sphingomyelin, lactate, gluconate, adenosine, 2-methylbutyrylglycine and guanidinoacetate was trained using the cohort-1 data set and then tested on the cohort-2 data set. ROC curves with AUCs are displayed for the training set (A.) and the test set (B.).

As a test example of potential biomarker performance in a multi-analyte algorithm, palmitoyl sphingomyelin, lactate, gluconate, adenosine, 2-methylbutyrylglycine and guandinoacetate were chosen for algorithm training using the cohort-1 data set. These candidate biomarkers were chosen based on their fold differences and p-values in both cohort-1 and cohort-2. The algorithm derived from training on the cohort-1 data set was tested on the cohort-2 data set. AUCs and ROC curves for both the training and test set analysis are displayed in Fig. 7. Comparable AUCs were obtained for both cohorts, with AUC  = 0.81 for cohort-1 and 0.78 for cohort-2. Specificity values remained high, up to a sensitivity cutoff of around 0.5, in both cohorts. The performance observed using this algorithm does not infer future predictive value, since biomarkers used in the algorithm were pre-selected based on their tumor differentiation ability in both cohorts. This example illustrates that it is possible to derive an algorithm which segregates tumor from control urines in both of these specific cohorts.

## Discussion

Bladder cancer is a significant cause of morbidity and mortality with a high recurrence rate and need for frequent follow-up surveillance. Currently, monitoring for recurrence requires cystoscopy on a semi-routine basis, typically until an extensive disease free period has transpired. A more facile, less invasive diagnostic methodology would be advantageous for patient management and might increase follow-up surveillance compliance. Measurement of urine metabolites may provide a companion diagnostic method which could facilitate the monitoring of bladder cancer recurrence and perhaps also contribute to primary diagnosis.

Recent metabolomic studies have proven valuable in identifying cancer biomarkers and in gaining insights into the role of metabolic reprogramming in the initiation and progression of malignancies. Metabolic reprogramming in tumor cells is a common phenomenon and is now recognized as an emerging hallmark of cancer [28]. Changes in metabolite levels resulting from tumor metabolic reprogramming can offer unique opportunities for biomarker discovery. For example, 2-hydroxyglutarate is increased in gliomas, multiple myeloma and colon cancer [29] and elevated sarcosine is associated with prostate and colorectal cancer [30], [31]. Metabolites associated with tumor cell metabolic reprogramming or perhaps tumor-stromal interactions might be anticipated to display a change in levels not only in the tumor tissue itself, but also in matrices such as blood or urine which support uptake or excretion of biochemicals connected to tumor growth or invasion. Several investigations have reported on the usefulness of metabolite biomarkers to diagnose, stratify and monitor cancer patients [31]–[34].

The present study profiled 430 urine samples from two cohorts of subjects, with known positive or negative BCA diagnoses and as such, represents the most comprehensive screening for bladder cancer urinary metabolite biomarkers to date. Previous studies have measured a limited number of metabolites in urine (typically less than 25). The non-targeted UPLC/mass spectrometry based technology platform employed in this study facilitates the identification and relative quantitation of >500 chemical compounds, in urine samples, greatly expanding the number of potential biomarker candidates over those previously described. 25 metabolites were selected from cohort 1 for evaluation in the independent cohort 2 data set. The 25 biochemicals identified as candidate biomarkers covered a broad range of metabolic pathways. While the 25 candidate biomarker set contained both increased and decreased metabolites - chosen to best explore multi-analyte predictive algorithms - hypotheses for increased urine metabolites in BCa are more easily generated than hypotheses for decreased metabolite levels. Increased metabolites could derive from tumor metabolites secreted into the urine or from breakdown or alteration of non-malignant tissue caused by the invasion of tumor through the epithelium wall. Inflammatory responses resulting from the presence of tumor might also result in increased levels of metabolites. Declines in metabolites might be caused a lower rate of metabolite excretion by tumor cells relative to normal epithelium or by an uptake of metabolites from the urine into the tumor or adjacent tissue. Changes in systemic metabolism caused by factors released by bladder tumors or remodeled adjacent tissues and subsequent urinary excretion, might also cause changes in urine metabolite levels, both increases and decreases. 25 metabolites were selected as biomarker candidates from the cohort 1 data set based on multiple criteria. The random forest analysis testing the 25 metabolites against the cohort 2 data set illustrated that a subset of the 25 stood out as better performers. Palmitoyl sphingomyelin, lactate, adenosine and succinate had the highest predictive value, with other metabolites displaying a range of reduced values. One possible explanation for the weaker performance of many of the cohort 1 candidate biomarkers might be that cohort 1 bladder cancer positive urine samples were derived from a higher percentage of subjects with high stage/high grade tumors than those present in subjects from cohort 2. It is also possible that many of the cohort 1 candidate biomarkers were false positives resulting from unique features of that particular sample population.

The 25 cohort 1 candidate biomarkers represent a diverse set of metabolic pathways – in part because pathway diversity was a filter for selecting the set of 25 from >200 metabolites with statistically significant differences comparing cohort 1 BCa positive urines from the combined group of all negative controls. Several pathways represented by the candidate metabolites were of particular interest. A major metabolic hallmark of cancer is the frequently observed shift from oxidative phosphorylation to a greater dependence on glucose metabolism through glycolysis, even under aerobic conditions (Warburg metabolism) [35]. While many different mechanisms are believed to contribute to this switch in metabolic activity, outcomes include increased uptake and consumption of glucose, increased lactate production and excretion, elevated citrate production, and increased pentose phosphate pathway (PPP) activity. Upregulating these pathways provides energy, fatty acid, nucleotide biosynthesis, and NADPH generation [36], [37]. Lactate levels were significantly increased in the urine samples from bladder cancer patients in cohorts 1 and 2 and may be an indication of increased glycolysis in BCa cells. In addition to lactate, β-hydroxypyruvate, which has not been previously linked to tumor metabolism, was significantly elevated in urine of primary bladder cancer subjects. β-hydroxypyruvate can be connected to glycolysis though its formation via the serine-pyruvate transaminase reaction or its derivation from the glycolysis intermediate 3-phosphoglycerate [38].

Three metabolites associated with lipid metabolism, palmitoyl sphingomyelin, phosphocholine and arachidonate (cohort 2 only) were significantly altered in urine of BCA subjects. This was somewhat surprising since; in general, lipids are not abundantly secreted in the urine. Sphingomyelin is a major component of the outer plasma membranes of cells [39]. Choline phosphate is a component of both glycerophospholipids and sphingomyelin. Cleavage of sphingomyelin, by neutral sphingomyelinases, results in the formation of both choline phosphate and ceramide [40]. Increased levels of palmitoyl sphingomyelin and choline phosphate, in the urine of BCA subjects, may reflect a relatively higher tumor cell proliferation rate and increased lipid membrane remodeling. If this is occurring, there may be an increased shedding of palmitoyl sphingomyelin into the urine of bladder cancer subjects and subsequent sphingomyelinases activity in the urine may result in increased choline phosphate. Another possible explanation for elevated palmitoyl sphingomyelin levels may be increased shedding of microvesicles by bladder tumors. The elevation of arachidonate may be of associated with increased liberation of free fatty acids from phospholipids either in the tumor or in adjacent tissue. Liberated arachidonate has a potential to play a role in inflammatory processes [41].

Increased branched chain amino acids (BCAAs) catabolism can provide an energy source for cells through anaplerotic mechanisms which feed the TCA cycle [42]. Levels of the three BCAAs leucine, isoleucine and valine were all higher in cohort 1 BCa urines relative to the normal and BCa history controls. The BCAA associated catabolite 2-methylbutrylglycine was lower in cohort 1 BCa urines relative to all control groups. Elevation of BCAAs may suggest an increased mobilization of amino acids to support the TCA cycle through anaplerotic reactions [43].

Three metabolites which can be indicators of mitochondrial TCA cycle activity, 3-hydroxybutyrate [BHBA], 2-hydroxybutyrate [AHB], and acetylcarnitine were elevated in cohort 1 BCa urines. Elevation of these metabolites can be an indication of decreased carbon flow into the TCA cycle or excess production of acetyl-CoA (or propionyl-CoA in the case of AHB increases) [44]–[46]. Increases in fatty acid β-oxidation or glycolysis might lead to excess acetyl-CoA production, which can be shunted into BHBA or acetylcarnitine if the TCA cycle does not take up all the acetyl-CoA that is synthesized. A shift toward decreased reliance on mitochondrial oxidative phosphorylation, frequently observed in tumor cells [47], might also contribute to BHBA, AHB and acetylcarnitine increases.

Tumor development and proliferation is dependent, in part, on metabolic reprogramming to support the increased energy and biosynthetic demands of the malignant phenotype. It is reasonable to hypothesize that these metabolic changes may result in unique metabolite signatures in the urines of subjects with bladder cancer, relative to urines from non-cancer controls. While the current study does not define an optimal set of biomarkers for BCa detection, this discovery study does demonstrate the possibility of employing urine metabolites as non-invasive biomarkers to complement existing diagnostic methods and provide improvements to bladder cancer patient monitoring and care. It is encouraging to note that a majority of the biomarker candidates identified were capable of distinguishing NMIBC tumor urines from controls, as it will be important that any bladder cancer test be capable of detecting high grade NMIBC tumors when used to support patient surveillance. Identification of a set of candidate biomarkers will allow the pursuit of metabolite panels which best predict the probability of BCa recurrence and which may also provide value in primary diagnosis.

## Conclusions

An unbiased global metabolomic profiling of urine samples from subjects with and without bladder cancer identified a set of candidate biomarkers for bladder cancer. A subset of metabolites displayed statistically significant differences in cancer vs. non-cancer urines in both of two independent sample cohorts. Some urine metabolite differences may reflect a reprogramming of glycolysis and lipid metabolism in tumor tissue. Future quantitative targeted assays based on the identified biomarker candidates will be required to validate the predictive value of these metabolites. These results demonstrate the potential of utilizing urine metabolites as a non-invasive test for bladder cancer and offer the possibility of a much needed adjunct to cystoscopy for detection and management of recurrent disease.

## Supporting Information

S1 Table Cohort 1 heatmap of all measured named metabolites. A Wilcoxon test was performed for each group comparison for all metabolites measured. Relative metabolite ratios are presented for BCa positive versus all control groups and also the combined control data set. Cell colors represent: dark red  =  higher in BCa at p≤0.05 significance; dark green  =  lower in BCa at p<0.05; light red  =  higher in BCa at p≤0.1; light green  =  lower in BCa at p<0.1.
(PDF)

S2 Table Cohort 2 heatmap of all measured named metabolites. Relative metabolite ratios and Wilcoxon statistical significance determinations as described for S1 Table.
(PDF)
