# Supplementary material for: Bladder Cancer Biomarker Discovery Using Global Metabolomic Profiling of Urine
Source: PLoS One. 2014 Dec 26;9(12):e115870. doi: 10.1371/journal.pone.0115870 (PMC4277370; doi:10.1371/journal.pone.0115870)
Supplement: S1 Table — Cohort 1 heatmap of all measured named metabolites. A Wilcoxon test was performed for each group comparison for all metabolites measured. Relative metabolite ratios are presented for BCa positive versus all control groups and also the combined control data set. Cell colors represent: dark red = higher in BCa at p≤0.05 significance; dark green = lower in BCa at p<0.05; light red = higher in BCa at p≤0.1; light green = lower in BCa at p<0.1. (PDF) [file pone.0115870.s001.pdf]

Supplementary Table S1 Cohort 1 heatmap of all measured named metabolites. Relative metabolite ratios are presented for Bca positive versus all control groups and also the combined control data set. Metabolites are grouped by their associated metabolism sub pathway. Cell colors represent: dark red = higher in BCa at p ≤ 0.05 significance; dark green = lower in BCa at p < 0.05; light red = higher in BCa at p ≤ 0.1; light green = lower in BCa at p < 0.1. The platform column designates the chromatography/MS platform the measurements were derived from. Pubchem reference numbers for the individual metabolites are given.

Sub Pathway	Biochemical Name	Platform	PUBCHEM	Fold Difference				Statistical Values							
								(BCA)/(Normal)		(BCA)/(History)		(BCA)/(Hematuria)		(BCA)/(All Ctrls)	
				BCa Normal	BCa History	BCa Hemturia	BCa All CTRLs	p-value	q-value	p-value	q-value	p-value	q-value	p-value	q-value
Glycine, Serine and Threonine Metabolism	glycine	GC/MS	750	1.13	0.86	0.75	0.89	0.596	0.1864	0.5559	0.2155	0.0554	0.0473	0.527	0.1625
	N-acetylglycine	GC/MS	10972	1.8	1.62	1.27	1.53	0.193	0.0787	0.1633	0.0858	0.4159	0.2072	0.1481	0.0584
	sarcosine (N-Methylglycine)	GC/MS	1088	0.79	0.78	0.75	0.78	0.0186	0.0123	0.0103	0.0087	0.0072	0.0094	0.0028	0.0021
	dimethylglycine	GC/MS	673	0.89	0.92	0.93	0.91	0.3802	0.1317	0.8086	0.2811	0.0792	0.0611	0.331	0.113
	serine	GC/MS	5951	0.99	0.96	0.76	0.89	0.542	0.1732	0.4728	0.1936	0.0375	0.0348	0.2313	0.0853
	N-acetylserine	LC/MS pos	65249	1.48	1.16	0.85	1.11	0.0021	0.002	0.1481	0.0792	0.7618	0.3264	0.0648	0.0294
	beta-hydroxypyruvate	GC/MS	964	1.79	1.94	2.61	2.06	0.0097	0.0071	0.0134	0.011	0.0037	0.0057	0.002	0.0015
	threonine	GC/MS	6288	1	0.94	0.81	0.91	0.6813	0.2072	0.8827	0.2972	0.0231	0.0238	0.3748	0.1242
	N-acetylthreonine	LC/MS neg	152204	0.96	0.95	0.78	0.89	0.8921	0.2556	0.5237	0.2086	0.003	0.0047	0.2209	0.0821
	allo-threonine	GC/MS	99289	0.68	0.76	0.71	0.72	0.0002	0.0003	0.0068	0.0062	0.0025	0.0042	0.0002	0.0002
	homoserine	GC/MS	12647	0.92	1.02	1.01	0.98	0.0267	0.0165	0.0287	0.0208	0.0436	0.0391	0.0111	0.0066
	2-phenylglycine	GC/MS	3866	1.62	1.3	1.64	1.51	0.0458	0.0256	0.3354	0.1497	0.0123	0.0145	0.0468	0.0226
Alanine and Aspartate Metabolism	alanine	GC/MS	5950	0.92	0.86	0.69	0.81	0.2261	0.0891	0.0703	0.0422	0.0004	0.0009	0.0162	0.0094
	N-acetylalanine	LC/MS neg	88064	0.82	0.72	0.87	0.8	0.0855	0.0412	0.049	0.0317	0.3064	0.1706	0.0502	0.024
	aspartate	GC/MS	5960	1.26	1.16	1.19	1.2	0.4291	0.1428	0.9463	0.3115	0.41	0.2047	0.9116	0.2464
	asparagine	GC/MS	6267	0.83	0.78	0.73	0.78	0.1061	0.048	0.0116	0.0097	0.0009	0.0017	0.0043	0.003
	N-acetylasparagine	LC/MS pos	99715	0.92	0.84	0.8	0.85	0.3015	0.1101	0.0945	0.0543	0.0057	0.0079	0.0417	0.021
	N-acetylaspartate (NAA)	LC/MS pos	65065	1.12	1.09	0.94	1.04	0.629	0.1943	0.3553	0.1563	0.1955	0.1237	0.7772	0.2186
Glutamate Metabolism	glutamate	GC/MS	611	1.6	1.48	1.15	1.38	0.0914	0.0435	0.2643	0.1238	0.9182	0.3657	0.2313	0.0853
	glutamine	LC/MS pos	5961	1.65	1.28	0.96	1.24	3.85E-05	0.0001	0.006	0.0055	0.335	0.1804	0.001	0.0009
	N-acetylglutamate	LC/MS pos	70914	0.88	0.88	0.83	0.86	0.0707	0.0357	0.2487	0.1182	0.0185	0.0198	0.0491	0.0236
	N-acetylglutamine	LC/MS neg	182230	0.9	0.85	0.78	0.84	0.443	0.1465	0.4518	0.1901	0.0194	0.0205	0.1769	0.0684
	N-acetyl-aspartyl-glutamate (NAAG)	LC/MS pos	5255	1.2	1.33	1.12	1.21	0.5985	0.187	0.3651	0.1602	0.3904	0.1985	0.6778	0.196
	gamma-aminobutyrate (GABA)	GC/MS	119	0.55	0.76	0.68	0.65	0.0001	0.0002	0.0138	0.0112	0.0074	0.0096	0.0004	0.0004
	N-methylglutamate	GC/MS	439377	0.97	0.78	0.73	0.81	0.0016	0.0016	0.0003	0.0004	0.0004	0.0009	4.22E-05	0.0001
	pyroglutamine*	LC/MS pos	134508	0.93	0.92	0.82	0.89	0.1945	0.0791	0.4198	0.1795	0.027	0.0271	0.1209	0.049
	citramalate	GC/MS	1081	0.87	0.76	0.89	0.84	0.037	0.0216	0.0178	0.0139	0.0211	0.022	0.0076	0.0048
Histidine Metabolism	histidine	LC/MS neg	6274	1.33	1.09	0.94	1.1	0.0029	0.0026	0.2975	0.1358	0.5698	0.2638	0.124	0.0502
	N-acetylhistidine	LC/MS neg	75619	0.92	0.91	0.78	0.87	0.444	0.1466	0.257	0.1212	0.0055	0.0077	0.0958	0.0409
	1-methylhistidine	GC/MS	92105	0.55	0.71	0.61	0.62	0.0001	0.0002	0.0069	0.0062	0.0332	0.0315	0.0005	0.0005
	3-methylhistidine	LC/MS neg	64969	0.67	0.72	0.67	0.69	0.0115	0.0083	0.0208	0.0158	0.0482	0.0427	0.0065	0.0042
	hydantoin-5-propionic acid	LC/MS pos	782	0.98	0.98	1	0.99	0.8225	0.2393	0.8042	0.2801	0.7372	0.3188	0.907	0.2454
	trans-urocanate	LC/MS pos	736715	1.25	1.41	1.26	1.3	0.6086	0.1894	0.5655	0.2175	0.6254	0.282	0.956	0.2549
	cis-urocanate	LC/MS pos	1549103	1.09	0.98	1.16	1.07	0.3532	0.1242	0.9417	0.3109	0.3054	0.1705	0.4848	0.1521
	imidazole propionate	LC/MS pos	70630	1.4	1.48	1.16	1.34	0.0079	0.006	0.0001	0.0002	0.1298	0.0901	0.0007	0.0006
	imidazole lactate	LC/MS pos	440129	0.9	0.94	0.94	0.92	0.1355	0.0593	0.6291	0.2355	0.3011	0.1688	0.2586	0.0932
	1-methylimidazoleacetate	LC/MS pos	75810	1.03	1.07	0.97	1.03	0.392	0.1351	0.6009	0.2281	0.1724	0.1129	0.8337	0.2305
Lysine Metabolism	lysine	GC/MS	5962	0.65	0.36	0.54	0.48	0.1184	0.0528	0.1269	0.0697	0.0783	0.0608	0.0593	0.0275
	N2-acetyllysine	LC/MS pos	92907	0.77	0.78	0.8	0.78	0.0132	0.0094	0.0143	0.0115	0.0107	0.0129	0.0033	0.0024
	N6-acetyllysine	LC/MS pos	92832	0.82	0.96	0.81	0.86	0.0111	0.0081	0.4484	0.1888	0.0046	0.0067	0.0281	0.0151
	N-6-trimethyllysine	GC/MS	440120	0.77	0.84	0.7	0.77	0.157	0.0673	0.342	0.1518	0.0284	0.0283	0.0962	0.0409
	2-aminoadipate	GC/MS	469	0.65	0.84	0.64	0.7	0.0004	0.0005	0.0627	0.0387	0.0017	0.003	0.0012	0.001
	glutarate (pentanedioate)	GC/MS	743	0.72	0.83	0.81	0.79	0.0138	0.0096	0.0672	0.0411	0.0381	0.0351	0.013	0.0076
	glutarylcarnitine (C5)	LC/MS pos	71464488	0.96	1.1	1.02	1.02	0.6523	0.2001	0.1822	0.0927	0.8747	0.3568	0.3725	0.1235
	3-hydroxyglutarate	GC/MS	181976	0.7	0.78	0.78	0.75	0.0092	0.0068	0.0483	0.0316	0.0545	0.047	0.0102	0.0061
	pipecolate	LC/MS pos	849	0.61	0.38	0.67	0.52	0.4307	0.1432	0.0597	0.0372	0.0102	0.0124	0.0466	0.0225
	5-aminovalerate	GC/MS	138	1.52	1.69	1.41	1.53	0.0016	0.0016	0.0036	0.0036	0.0009	0.0018	0.0003	0.0003
Phenylalanine and Tyrosine Metabolism	phenylalanine	LC/MS pos	6140	1.04	1	0.88	0.97	0.8098	0.2365	0.9031	0.3023	0.1153	0.0819	0.6773	0.196
	N-acetylphenylalanine	LC/MS neg	74839	0.59	0.5	0.86	0.62	0.0001	0.0002	2.97E-05	0.0001	0.1998	0.1258	0.0001	0.0001
	phenyllactate (PLA)	LC/MS neg	3848	0.59	0.7	0.78	0.68	0.2255	0.089	0.269	0.1259	0.0228	0.0237	0.0929	0.04
	4-hydroxyphenylacetate	GC/MS	127	0.47	0.61	0.49	0.52	3.94E-07	2.04E-06	1.33E-07	5.80E-07	3.75E-06	2.76E-05	2.38E-09	1.04E-08
	3-hydroxyphenylacetate	GC/MS	12122	0.28	0.37	0.35	0.33	2.98E-11	2.98E-09	3.52E-10	4.45E-09	3.32E-06	2.58E-05	1.25E-12	2.12E-11
	phenylacetylglycine	LC/MS pos	68144	0.58	0.52	0.51	0.54	0.0033	0.0029	0.0001	0.0001	2.34E-05	0.0001	1.24E-05	1.86E-05
	phenylacetylglutamine	LC/MS pos	92258	0.71	0.7	0.69	0.7	1.70E-05	4.19E-05	6.76E-06	1.59E-05	0.0001	0.0002	3.64E-07	7.68E-07
	tyrosine	LC/MS pos	6057	1.13	1.05	0.9	1.02	0.1566	0.0672	0.3015	0.1371	0.4848	0.2313	0.3802	0.1254
	N-acetyltyrosine	LC/MS neg	68310	0.66	0.6	0.97	0.72	0.0024	0.0022	0.0001	0.0001	0.0542	0.0468	0.0002	0.0002
	tyramine	LC/MS pos	5610	0.68	0.76	0.56	0.66	0.0001	0.0002	0.0125	0.0103	9.42E-06	0.0001	0.0001	0.0001
	4-hydroxyphenylpyruvate	LC/MS neg	979	0.94	0.87	1.06	0.95	0.9336	0.2632	0.323	0.1457	0.9481	0.3734	0.6315	0.186
	3-(4-hydroxyphenyl)lactate	LC/MS neg	9378	0.74	0.6	1.3	0.79	0.074	0.0368	0.695	0.2519	0.4668	0.2255	0.2653	0.0952
	phenol sulfate	LC/MS neg	74426	0.94	0.81	0.8	0.85	0.0211	0.0135	0.0134	0.011	0.0086	0.0108	0.0037	0.0026
	p-cresol sulfate	LC/MS neg	4615423	0.48	0.53	0.61	0.54	7.11E-09	1.05E-07	8.96E-08	4.23E-07	2.35E-05	0.0001	6.59E-10	3.88E-09
	o-cresol sulfate	LC/MS neg	11615528	1.47	0.78	0.98	1.01	0.4049	0.1361	0.0428	0.0288	0.6324	0.2843	0.5812	0.1758
	L-methyldopa	LC/MS pos	38853	1	1	1	1			0.4633	0.1906			0.6237	0.1844
	dopamine	GC/MS	681	0.58	0.77	0.82	0.71	0.0203	0.0131	0.1217	0.0674	0.0391	0.0357	0.0223	0.0124
	vanillylmandelate (VMA)	GC/MS	1245	0.91	0.85	0.98	0.91	0.3477	0.1224	0.0202	0.0155	0.2463	0.148	0.062	0.0286
	3-methoxytyrosine	LC/MS pos	1670	1	0.66	1.95	0.99	0.1299	0.0575	0.2405	0.1153	0.7657	0.3274	0.2708	0.0968
	3,4-dihydroxyphenylacetate	GC/MS	547	0.46	0.57	0.64	0.55	1.70E-05	4.19E-05	0.0012	0.0014	0.0005	0.0011	1.61E-05	2.30E-05
	vanilpyruvate	LC/MS neg	14124	0.51	0.53	1.4	0.66	0.6255	0.1936	0.1187	0.066	0.26	0.1538	0.795	0.2221
	homovanillate (HVA)	LC/MS neg	1738	0.77	0.72	0.79	0.76	0.0567	0.0307	0.0488	0.0317	0.0021	0.0035	0.0087	0.0054
	homovanillate sulfate	LC/MS neg	29981063	0.85	0.69	0.99	0.83	0.2658	0.1002	0.0396	0.027	0.0919	0.0683	0.0508	0.0243
	3-methoxy-4-hydroxyphenylglycol	GC/MS	10805	0.96	0.99	1.07	1	0.4124	0.1379	0.273	0.1269	0.6795	0.3006	0.3211	0.1102
	gentisate	LC/MS neg	3469	0.12	0.21	0.21	0.17	1.00E-14	2.89E-12	7.71E-13	3.60E-11	4.26E-11	8.93E-09	0.00E+00	2.00E-15
	phenylpropionylglycine	LC/MS neg	152323	0.5	0.47	0.47	0.48	2.41E-06	8.94E-06	0.0001	0.0001	1.51E-05	0.0001	1.36E-06	2.53E-06
	2-(4-hydroxyphenyl)propionate	GC/MS	102526	0.4	0.67	0.3	0.41	0.0713	0.0358	0.7712	0.2723	0.1068	0.0775	0.1864	0.0712
	3-(3-hydroxyphenyl)propionate	GC/MS	91	0.47	0.27	0.57	0.39	0.0015	0.0015	7.82E-06	1.80E-05	0.0017	0.003	1.43E-05	2.10E-05
	2-hydroxyphenylacetate	GC/MS	11970	0.89	0.93	0.87	0.9	0.2825	0.1047	0.5196	0.2072	0.2354	0.1433	0.2764	0.0982
	tryptophan	LC/MS pos	6305	1.32	1.29	1.04	1.2	0.0011	0.0012	0.0005	0.0006	0.5834	0.268	0.0014	0.0011
	N-acetyltryptophan	LC/MS neg	700653	0.48	0.45	0.82	0.55	0.0014	0.0014	4.11E-05	0.0001	0.4673	0.2255	0.0004	0.0004
	indolelactate	LC/MS pos	92904	0.78	0.81	1	0.85	0.0341	0.0202	0.0967	0.0555	0.3503	0.1857	0.0526	0.0249
	indoleacetate	LC/MS pos	802	1.06	1.03	0.9	0.99	0.8863	0.2542	0.8623	0.2931	0.7278	0.315	0.8968	0.2437
	3-indoxyl sulfate	LC/MS neg	10258	0.51	0.54	0.56	0.54	4.79E-08	4.79E-07	8.42E-07	2.59E-06	5.00E-06	3.23E-05	3.02E-09	1.30E-08

Sub Pathway	Biochemical Name	Platform	PUBCHEM	Fold Difference				Statistical Values							
								(BCA)/(Normal)		(BCA)/(History)		(BCA)/(Hematuria)		(BCA)/(All Ctrls)	
				BCa Normal	BCa History	BCa Hemturia	BCa All CTRLs	p-value	q-value	p-value	q-value	p-value	q-value	p-value	q-value
Tryptophan Metabolism	kynurenine	LC/MS pos	161166	2.17	1.71	1.93	1.92	0.0002	0.0003	0.0119	0.0099	0.0511	0.0446	0.001	0.0008
	kynurenate	LC/MS neg	3845	0.91	0.83	0.91	0.88	0.4042	0.1361	0.1306	0.0715	0.0891	0.0669	0.1155	0.0472
	anthranilate	LC/MS pos	227	0.81	0.73	0.9	0.8	0.0619	0.0323	0.0483	0.0316	0.088	0.0663	0.0323	0.017
	3-hydroxykynurenine	LC/MS pos	89	1.3	0.79	1.27	1.07	0.094	0.0444	0.0282	0.0205	0.0574	0.0481	0.021	0.0117
	3-hydroxyanthranilate	LC/MS pos	86	1.14	1.19	1.2	1.18	0.6956	0.211	0.2552	0.1207	0.3985	0.2008	0.3337	0.1138
	xanthurenate	LC/MS pos	5699	0.58	0.66	0.69	0.64	2.21E-07	1.33E-06	0.0002	0.0003	0.0002	0.0004	5.99E-07	1.21E-06
	picolinate	LC/MS pos	1018	0.81	0.92	0.97	0.89	0.0377	0.0219	0.975	0.3192	0.8173	0.3423	0.3624	0.1212
	5-hydroxyindoleacetate	GC/MS	1826	0.66	0.85	0.84	0.77	0.0269	0.0166	0.273	0.1269	0.1389	0.0944	0.0645	0.0294
	tryptophan betaine	LC/MS pos	442106	0.83	1.02	0.8	0.88	0.1657	0.0706	0.4247	0.1805	0.5118	0.2409	0.7358	0.2101
	C-glycosyltryptophan*	LC/MS pos		1.02	0.98	0.99	1	0.5663	0.1792	0.2717	0.1266	0.4632	0.2246	0.3232	0.1106
Leucine, Isoleucine and Valine Metabolism	leucine	LC/MS pos	6106	1.34	1.27	1.19	1.26	0.0102	0.0074	0.0078	0.0069	0.3011	0.1688	0.0082	0.0051
	N-acetylleucine	LC/MS pos	70912	0.95	1.01	0.84	0.93	0.7364	0.2205	0.7255	0.2596	0.4215	0.2092	0.8511	0.2337
	4-methyl-2-oxopentanoate	LC/MS neg	70	1.67	1.94	1.32	1.6	0.007	0.0054	0.0003	0.0004	0.1104	0.0794	0.0008	0.0007
	isovalerylglycine	LC/MS neg	546304	0.56	0.53	0.49	0.53	2.85E-06	1.04E-05	5.90E-09	4.64E-08	4.86E-09	2.27E-07	1.21E-10	8.75E-10
	isovalerylcarnitine	LC/MS pos	6426851	0.97	1.22	1.2	1.12	0.9265	0.2622	0.5501	0.214	0.8003	0.3377	0.6825	0.197
	3-methylcrotonylglycine	LC/MS pos	169485	0.59	0.52	0.58	0.56	0.0015	0.0015	4.34E-07	1.52E-06	2.28E-05	0.0001	6.55E-07	1.30E-06
	beta-hydroxyisovalerate	LC/MS neg	69362	0.92	1.1	0.9	0.97	0.0663	0.0341	0.3055	0.1387	0.0726	0.0571	0.4663	0.1479
	beta-hydroxyisovaleroylcarnitine	LC/MS pos		0.94	1.04	0.98	0.98	0.1142	0.051	0.9372	0.3103	0.5297	0.2488	0.3975	0.1293
	3-methylglutarylcarnitine (C6)	LC/MS pos	128145	0.94	0.92	1.22	1.01	0.2612	0.0988	0.5675	0.218	0.9302	0.3687	0.4804	0.1517
	3-methylglutaconate	LC/MS pos	1551553	0.79	0.88	0.96	0.87	0.0027	0.0024	0.045	0.0297	0.0702	0.0561	0.0067	0.0043
	3-methylglutarate	GC/MS	12284	0.88	1.01	1.11	0.99	0.0413	0.0238	0.2446	0.1167	0.396	0.2001	0.1023	0.043
	alpha-hydroxyisovalerate	GC/MS	99823	2.39	1.04	2.11	1.61	0.9645	0.2686	0.3105	0.1408	0.767	0.3276	0.6673	0.1941
	methylsuccinate	LC/MS pos	10349	1.05	1.02	0.95	1	0.7053	0.2135	0.6808	0.2491	0.4785	0.2291	0.7959	0.2221
	isoleucine	LC/MS pos	6306	1.41	1.36	1.23	1.33	0.0042	0.0035	0.0058	0.0054	0.2463	0.148	0.0044	0.0031
	N-acetylisoleucine	LC/MS pos	2802421	0.81	0.93	0.81	0.85	0.0118	0.0085	0.7678	0.2717	0.0197	0.0208	0.0699	0.0314
	3-methyl-2-oxovalerate	LC/MS neg	47	1.65	1.59	1.12	1.41	0.006	0.0048	0.0019	0.0021	0.6182	0.2794	0.0054	0.0036
	2-methylbutyrylcarnitine (C5)	LC/MS pos	6426901	1	1.07	0.96	1.01	0.6342	0.1957	0.487	0.1969	0.3849	0.1969	0.9286	0.25
	2-methylbutyrylglycine	LC/MS neg	193872	0.68	0.65	0.63	0.65	0.0009	0.001	3.03E-05	0.0001	2.69E-06	2.13E-05	2.14E-06	3.83E-06
	tiglyl carnitine	LC/MS pos	22833596	0.87	0.94	0.93	0.91	0.0386	0.0224	0.3363	0.1498	0.2895	0.1652	0.1087	0.0451
	tigloylglycine	LC/MS pos	6441567	0.79	0.73	0.77	0.76	0.0942	0.0444	0.0102	0.0087	0.0027	0.0044	0.005	0.0034
	3-hydroxy-2-ethylpropionate	GC/MS	188979	1.57	1.72	1.51	1.59	0.3608	0.1257	0.0392	0.0267	0.4016	0.2015	0.1022	0.043
	ethylmalonate	LC/MS pos	11756	1.06	1.28	0.87	1.05	0.1875	0.077	0.0174	0.0137	0.5989	0.273	0.119	0.0484
	valine	LC/MS pos	6287	1.34	1.24	1.18	1.25	0.0027	0.0024	0.0142	0.0114	0.2363	0.1434	0.0059	0.0038
	N-acetylvaline	LC/MS neg	66789	0.84	0.92	0.84	0.87	0.0311	0.0187	0.6558	0.2427	0.0674	0.0543	0.1095	0.0454
	isobutyrylcarnitine	LC/MS pos	168379	0.77	0.89	0.85	0.84	0.0001	0.0002	0.0028	0.0029	0.0004	0.001	0.0001	0.0001
	isobutyrylglycine	LC/MS pos	10855600	0.61	0.61	0.64	0.62	2.01E-07	1.23E-06	3.82E-08	2.02E-07	3.43E-06	2.61E-05	8.44E-10	4.78E-09
	3-hydroxyisobutyrate	GC/MS	87	0.91	1.09	0.92	0.96	0.1101	0.0496	0.8421	0.2894	0.1248	0.0878	0.2409	0.088
Methionine, Cysteine, SAM and Taurine Metabolism	methionine	GC/MS	6137	0.86	0.92	0.91	0.89	0.0701	0.0356	0.6851	0.2496	0.0587	0.0489	0.1437	0.0572
	N-acetylmethionine	LC/MS neg	448580	0.95	1.11	1.01	1.02	0.1969	0.0799	0.6781	0.2486	0.2491	0.1488	0.3047	0.1063
	S-adenosylhomocysteine (SAH)	LC/MS neg	439155	0.9	0.94	0.95	0.93	0.303	0.1105	0.5419	0.2123	0.2736	0.16	0.3042	0.1063
	cystathionine	GC/MS	439258	0.68	0.74	0.61	0.67	0.0579	0.031	0.0396	0.027	0.0331	0.0315	0.0179	0.0102
	2-aminobutyrate	GC/MS	439691	1.4	1.4	1.17	1.31	0.2317	0.0906	0.0748	0.0443	0.8746	0.3568	0.1584	0.062
	2-hydroxybutyrate (AHB)	GC/MS	440864	2.96	3.29	2.04	2.65	4.73E-06	1.51E-05	2.61E-07	9.82E-07	0.0001	0.0002	3.38E-08	1.02E-07
	cysteine	GC/MS	5862	0.92	0.92	1.04	0.96	0.5663	0.1792	0.1859	0.0939	0.6869	0.3026	0.3149	0.1086
	N-acetylcysteine	LC/MS neg	12035	0.66	0.61	0.59	0.62	0.0042	0.0035	0.0007	0.0008	0.0042	0.0061	0.0004	0.0004
	cystine	GC/MS	67678	0.35	0.22	0.46	0.31	0.0001	0.0002	0.0084	0.0073	0.0118	0.014	0.0004	0.0003
	S-methylcysteine	GC/MS	24417	0.93	1.04	0.86	0.94	0.1476	0.0636	0.7577	0.2684	0.19	0.1212	0.2699	0.0966
	taurine	GC/MS	1123	1.7	1.55	1.35	1.52	0.0446	0.0249	0.0735	0.0439	0.5781	0.2667	0.0643	0.0294
Urea cycle; Arginine and Proline Metabolism	arginine	GC/MS	232	0.39	0.14	0.61	0.27	0.0046	0.0038	5.24E-06	1.28E-05	0.0021	0.0035	2.08E-05	2.87E-05
	urea	GC/MS	1176	1.14	1.03	0.98	1.05	0.0707	0.0357	0.7298	0.2606	0.9143	0.3653	0.3573	0.1199
	ornithine	GC/MS	6262	0.91	0.49	0.85	0.69	0.9567	0.2673	0.4799	0.1954	0.293	0.1652	0.527	0.1625
	proline	LC/MS pos	145742	1.2	0.95	1.1	1.07	0.1753	0.0736	0.7755	0.2728	0.8237	0.3437	0.5383	0.1653
	homocitrulline	LC/MS pos	65072	0.98	1.08	0.91	0.98	0.427	0.1423	0.3392	0.1507	0.563	0.2609	0.9983	0.2634
	dimethylarginine (SDMA + ADMA)	LC/MS pos	123831	0.95	1.02	0.9	0.95	0.2374	0.0923	0.4536	0.1906	0.058	0.0486	0.1657	0.0645
	N-acetylarginine	LC/MS pos	67427	0.9	0.93	0.85	0.89	0.0611	0.032	0.2226	0.1085	0.0818	0.0626	0.0643	0.0294
	N-acetylproline	LC/MS neg	322640	0.71	0.65	0.69	0.68	0.0012	0.0012	0.0004	0.0005	0.0003	0.0008	0.0001	0.0001
	N-methyl proline	GC/MS	557	1.6	0.67	1.16	1	0.0014	0.0014	0.3193	0.1442	0.2739	0.16	0.0378	0.0193
	3-hydroxyproline	GC/MS	559314	0.8	0.66	0.83	0.76	0.0786	0.0384	0.0137	0.0111	0.1126	0.0805	0.0172	0.0099
	pro-hydroxy-pro	LC/MS pos	11673055	1.13	1.17	1.02	1.1	0.5687	0.1797	0.2534	0.12	0.8668	0.3561	0.3975	0.1293
Creatine Metabolism	creatine	LC/MS pos	586	0.31	0.45	0.35	0.36	0.0098	0.0072	0.0198	0.0152	0.002	0.0035	0.0021	0.0016
	creatinine	LC/MS pos	588	1.14	1	0.86	0.99	0.4838	0.1569	0.8399	0.2894	0.3687	0.1902	0.9355	0.2516
	N-carbamoylsarcosine	LC/MS pos	439375	0.86	0.8	0.85	0.84	0.0195	0.0127	0.004	0.0039	0.0056	0.0078	0.0016	0.0013
	guanidinoacetate	GC/MS	763	0.63	0.52	0.5	0.54	0.0005	0.0006	0.001	0.0011	0.0009	0.0018	0.0001	0.0001
Polyamine Metabolism	guanidine	GC/MS	3520	0.5	0.53	0.53	0.52	3.01E-05	0.0001	0.0001	0.0002	0.0025	0.0041	1.18E-05	1.77E-05
	putrescine	GC/MS	1045	1.22	1.25	0.61	0.92	0.0089	0.0066	0.027	0.0197	0.0115	0.0136	0.0043	0.003
	5-methylthioadenosine (MTA)	LC/MS pos	439176	1.07	1.09	0.98	1.05	0.3385	0.1197	0.145	0.0782	0.8393	0.3482	0.2432	0.0886
	N-acetylputrescine	LC/MS pos	122356	0.93	0.93	0.9	0.92	0.872	0.2513	0.5262	0.2092	0.7427	0.3201	0.634	0.1863
	4-acetamidobutanoate	LC/MS pos	18189	1	0.98	1.01	1	0.7598	0.2256	0.7471	0.2654	0.8432	0.3495	0.923	0.2487
Guanidino and Acetamido Metabolism	guanidinosuccinate	LC/MS pos	97856	1.58	1.11	1.48	1.36	0.0695	0.0355	0.478	0.1948	0.2986	0.1679	0.17	0.0659
Glutathione Metabolism	glutathione, reduced (GSH)	LC/MS pos	124886	7.25	9.27	6.62	7.56	0.0212	0.0136	3.79E-05	0.0001	0.0305	0.03	0.0002	0.0002
	glutathione, oxidized (GSSG)	LC/MS pos	65359	2.25	2.14	2.18	2.19	0.0036	0.0031	0.0017	0.0019	0.0056	0.0078	1.87E-05	2.59E-05
	cysteine-glutathione disulfide	LC/MS pos	4247235	2.29	2.07	2.53	2.28	0.3989	0.1353	0.9535	0.313	0.1511	0.101	0.4871	0.1523
	5-oxoproline	LC/MS neg	7405	0.85	0.88	0.85	0.86	0.1008	0.0466	0.0518	0.0331	0.0315	0.0306	0.0252	0.0137
Gamma-glutamyl Amino Acid	gamma-glutamylisoleucine*	LC/MS pos	14253342	0.89	0.96	0.83	0.89	0.1109	0.0498	0.507	0.2036	0.0131	0.0151	0.0941	0.0403
	gamma-glutamylleucine	LC/MS pos	151023	0.73	0.75	0.7	0.72	0.029	0.0176	0.033	0.0234	0.0057	0.0078	0.0065	0.0042
	gamma-glutamylphenylalanine	LC/MS pos	111299	1.49	1.16	1.17	1.25	0.0001	0.0001	0.0229	0.0172	0.0628	0.0512	0.0011	0.0009
	gamma-glutamylthreonine*	LC/MS pos		0.89	0.89	0.78	0.85	0.2816	0.1045	0.3986	0.172	0.008	0.0102	0.1077	0.045
	gamma-glutamyltyrosine	LC/MS pos	94340	0.74	0.77	0.73	0.75	0.0088	0.0065	0.0562	0.0353	0.0395	0.036	0.0098	0.006
	gamma-glutamylvaline	LC/MS pos	7015683	0.76	0.85	0.73	0.78	0.0017	0.0017	0.0945	0.0543	0.0001	0.0004	0.0015	0.0012
Dipeptide Derivative	carnosine	LC/MS neg	439224	0.52	0.39	0.33	0.4	0.0322	0.0192	0.0688	0.0415	4.03E-05	0.0001	0.0039	0.0027
	anserine	LC/MS neg	112072	0.23	0.14	0.23	0.19	0.0042	0.0035	0.0036	0.0036	0.0002	0.0005	0.0003	0.0003
	alanylalanine	GC/MS	5484352	0.74	0.8	0.94	0.82	0.0119	0.0085	0.0295	0.0213	0.539	0.2526	0.0238	0.0131
	aspartylaspartate	GC/MS	332965	0.58	0.7	0.72	0.66	0.0019	0.0018	0.0297	0.0215	0.0714	0.0567	0.0046	0.0032
	cyclo(gly-pro)	LC/MS pos	126154	1.18	1.09	1.09	1.12	0.0611	0.032	0.1752	0.0909	0.1369	0.0934	0.0649	0.0294
	cyclo(leu-pro)	LC/MS pos	7074739	0.97	0.99	0.92	0.96	0.1974	0.08	0.2158	0.1059	0.2392	0.145	0.143	0.0571
	glycylglycine	GC/MS	11163	0.89	0.88	0.97	0.91	0.1988	0.0801	0.0218	0.0164	0.2672	0.1574	0.0484	0.0233

Sub Pathway	Biochemical Name	Platform	PUBCHEM	Fold Difference				Statistical Values							
								(BCA)/(Normal)		(BCA)/(History)		(BCA)/(Hematuria)		(BCA)/(All Ctrls)	
				BCa / Normal	BCa / History	BCa / Hemturia	BCa / All CTRLs	p-value	q-value	p-value	q-value	p-value	q-value	p-value	q-value
Dipeptide	glycylproline	LC/MS pos	3013625	1.03	1.09	0.96	1.02	0.333	0.1182	0.6353	0.2371	0.3904	0.1985	0.6952	0.2
	phenylalanylphenylalanine	LC/MS pos	090;6993089	1.15	1.12	1.03	1.1	0.9195	0.2612	0.6657	0.2453	0.4929	0.2339	0.652	0.1909
	prolylglycine	LC/MS pos	076;6426709	0.88	0.7	0.76	0.77	0.1735	0.0732	0.0624	0.0386	0.2417	0.1461	0.0717	0.032
	pyroglutamylglutamine	LC/MS neg		0.77	0.84	0.86	0.82	0.0191	0.0125	0.0592	0.0369	0.2187	0.1352	0.0264	0.0142
	pyroglutamylglycine	LC/MS neg	152981	1.14	1.2	1.08	1.14	0.278	0.1036	0.1352	0.0733	0.6622	0.2942	0.192	0.0731
	pyroglutamylvaline	LC/MS neg	152416	0.96	0.9	0.94	0.93	0.2627	0.0992	0.0739	0.0441	0.4044	0.2026	0.1149	0.0471
Polypeptide	Ac-Ser-Asp-Lys-Pro-OH	LC/MS pos	4409396	0.61	0.86	1.52	0.87	0.0177	0.0118	0.5596	0.2157	0.2497	0.149	0.3309	0.113
Glycolysis, Gluconeogenesis, and Pyruvate Metabolism	1,5-anhydroglucitol (1,5-AG)	GC/MS	64960	1.28	1	0.82	1	0.059	0.0314	0.6127	0.2307	0.2805	0.1634	0.4891	0.1526
	glucose	GC/MS	79025	0.42	0.85	0.79	0.62	0.2289	0.0898	0.0268	0.0196	0.0679	0.0546	0.0351	0.0182
	glucose-6-phosphate (G6P)	GC/MS	5958	1.69	1.28	1.48	1.46	0.0294	0.0178	0.3444	0.1525	0.1545	0.103	0.0807	0.0354
	3-phosphoglycerate	GC/MS	724	1.05	0.87	1	0.97	0.0035	0.003	2.47E-05	0.0001	0.0106	0.0128	0.0001	0.0001
	phosphoenolpyruvate (PEP)	GC/MS	1005	2.4	2.02	2.58	2.31	0.1742	0.0733	0.7048	0.254	0.2107	0.1315	0.2781	0.0986
	pyruvate	GC/MS	1060	0.78	0.86	0.85	0.83	0.0422	0.0242	0.3151	0.1425	0.1724	0.1129	0.0894	0.0387
	lactate	GC/MS	612	3.14	3.13	1.41	2.23	2.96E-08	3.29E-07	6.02E-08	3.00E-07	0.0057	0.0078	1.22E-08	4.42E-08
Pentose Metabolism	ribulose	GC/MS	151261	0.94	1.06	0.91	0.97	0.1641	0.0701	0.9874	0.3217	0.0526	0.0458	0.2724	0.0971
	ribose	GC/MS	5779	0.81	1.02	0.76	0.85	0.6088	0.1894	0.5473	0.2137	0.2678	0.1575	0.8238	0.2285
	ribitol	GC/MS	6912	0.7	0.89	0.77	0.78	0.001	0.0011	0.135	0.0733	0.0005	0.0012	0.0023	0.0018
	xylulose	GC/MS	5289590	0.98	1.13	1.08	1.06	0.5492	0.1751	0.7105	0.2552	0.6795	0.3006	0.8652	0.2367
	xylonate	GC/MS	6602431	0.9	0.79	0.89	0.86	0.0287	0.0175	0.0018	0.002	0.0327	0.0313	0.0024	0.0018
	xylose	GC/MS	135191	1.07	1.06	0.86	0.99	0.1824	0.076	0.0681	0.0412	0.0142	0.016	0.0321	0.017
	xylitol	GC/MS	6912	0.52	0.94	1.04	0.76	0.1872	0.077	0.9531	0.313	0.4786	0.2291	0.4867	0.1523
	arabinose	GC/MS	66308	0.86	0.88	0.92	0.89	0.1859	0.0768	0.0792	0.0464	0.0695	0.0556	0.0546	0.0256
	threitol	GC/MS	169019	0.96	0.85	0.84	0.88	0.0016	0.0016	0.0005	0.0006	0.0005	0.0012	0.0001	0.0001
	arabitol	GC/MS	94154	0.84	0.9	0.85	0.86	0.0219	0.0139	0.036	0.0251	0.0244	0.0249	0.0093	0.0057
	glucono-1,5-lactone	GC/MS	7027	8.62	6.06	5.88	6.65	3.10E-05	0.0001	0.0012	0.0014	0.0128	0.0148	0.0001	0.0001
	fucose	GC/MS	19466	0.88	1.06	0.97	0.96	0.1015	0.0466	0.7755	0.2728	0.3988	0.2008	0.3013	0.1055
Glycogen Metabolism	maltose	GC/MS	10991489	2.96	5.1	2.41	3.16	0.1875	0.077	0.0104	0.0088	0.5321	0.2496	0.0446	0.022
Disaccharides and Oligosaccharides	lactose	GC/MS	84571	0.52	0.52	0.65	0.56	0.0016	0.0016	6.94E-06	1.62E-05	0.0052	0.0074	1.74E-05	2.44E-05
	3-sialyllactose	LC/MS neg	123914	0.89	0.93	0.83	0.88	0.4826	0.1568	0.2167	0.1061	0.1577	0.1048	0.1933	0.0734
	sucrose	LC/MS neg	5988	0.46	0.42	0.48	0.45	0.0019	0.0018	0.0001	0.0002	0.0012	0.0023	4.78E-05	0.0001
	galactinol	GC/MS	439451	0.47	0.48	0.67	0.52	0.001	0.0011	0.0001	0.0002	0.0365	0.0339	0.0002	0.0002
Fructose, Mannose and Galactose Metabolism	fructose	GC/MS	5984	0.55	0.46	0.51	0.51	0.0002	0.0003	1.25E-07	5.59E-07	1.57E-05	0.0001	7.98E-08	2.13E-07
	sorbose	GC/MS	441484	0.58	0.44	0.42	0.47	0.0002	0.0003	6.45E-06	1.52E-05	4.20E-06	2.92E-05	5.10E-07	1.04E-06
	sorbitol	GC/MS	5780	0.22	0.83	0.77	0.42	0.0009	0.001	0.0169	0.0133	0.0002	0.0005	0.0003	0.0003
	mannose	GC/MS	18950	0.67	0.71	0.87	0.74	0.0753	0.0374	0.0059	0.0054	0.0882	0.0663	0.0101	0.0061
	mannitol	GC/MS	6251	0.89	1.15	1.33	1.09	0.8239	0.2395	0.919	0.3062	0.6795	0.3006	0.9834	0.2599
	palatinitol	GC/MS	3034828	1.67	1.36	2.56	1.74	0.7029	0.213	0.2346	0.1132	0.7618	0.3264	0.4054	0.1315
	galactose	GC/MS	3037556	0.67	0.51	0.82	0.65	0.0183	0.0122	0.0003	0.0005	0.3081	0.1712	0.0025	0.0018
	galactitol (dulcitol)	GC/MS	11850	0.76	0.61	0.74	0.7	0.0072	0.0055	4.11E-06	1.01E-05	0.0014	0.0025	1.86E-05	2.58E-05
Nucleotide Sugar	UDP-glucuronate	GC/MS	17473	0.86	0.96	1.05	0.95	0.1534	0.0659	0.1434	0.0774	0.7016	0.3075	0.1592	0.0622
Aminosugar Metabolism	glucosamine	GC/MS	441477	0.46	0.44	0.4	0.43	0.0017	0.0017	0.0211	0.016	0.0035	0.0054	0.0012	0.001
	N-acetylneuraminate	GC/MS	439197	0.91	0.98	1.02	0.97	0.236	0.0919	0.2078	0.1032	0.4303	0.2126	0.1849	0.0709
	6-sialyl-N-acetyllactosamine	LC/MS pos	16212424	0.95	1.01	1.04	1	0.9783	0.2722	0.5694	0.2182	0.7849	0.3322	0.7079	0.2033
	erythronate*	GC/MS	2781043	0.84	0.89	0.92	0.88	0.0165	0.0113	0.0376	0.026	0.0792	0.0611	0.0127	0.0074
Advanced Glycation End-product	erythrulose	GC/MS	5460032	1.75	1.74	2.26	1.89	0.4039	0.1361	0.9461	0.3115	0.2543	0.151	0.5368	0.165
TCA Cycle	citrate	GC/MS	311	0.82	0.89	0.79	0.83	0.0126	0.009	0.6683	0.246	0.0449	0.0401	0.0755	0.0334
	cis-aconitate	LC/MS neg	643757	0.89	0.88	0.85	0.87	0.18	0.0752	0.0744	0.0442	0.0153	0.017	0.0342	0.0178
	isocitrate	LC/MS pos	1198	1	0.91	0.85	0.92	0.4883	0.1578	0.1063	0.0601	0.3634	0.1882	0.1792	0.0691
	alpha-ketoglutarate	GC/MS	51	1.35	1.48	1.21	1.34	0.2289	0.0898	0.0014	0.0016	0.851	0.3524	0.0436	0.0216
	succinylcarnitine	LC/MS pos		1.02	1.03	0.93	0.99	0.9294	0.2625	0.8086	0.2811	0.3251	0.1766	0.6606	0.193
	succinate	GC/MS	1110	0.65	0.51	0.6	0.58	1.05E-05	2.82E-05	2.88E-06	7.57E-06	0.0001	0.0002	1.64E-07	3.83E-07
	fumarate	GC/MS	444972	0.98	1	1.02	1	0.1719	0.0728	0.1937	0.0972	0.1031	0.0755	0.0972	0.0413
	malate	GC/MS	525	1.15	1.07	0.91	1.03	0.0366	0.0214	0.0153	0.0121	0.7091	0.3095	0.0317	0.0168
	trans-aconitate	LC/MS pos	444212	0.76	0.66	0.83	0.74	0.0087	0.0065	0.0033	0.0033	0.0257	0.0261	0.0017	0.0013
	itaconate (methylenesuccinate)	GC/MS	811	0.59	0.7	0.73	0.67	1.57E-06	6.29E-06	0.001	0.0011	0.0012	0.0023	7.59E-06	1.24E-05
	2-methylcitrate	GC/MS	439681	0.84	0.94	0.9	0.89	0.0993	0.0462	0.4083	0.1756	0.1514	0.1011	0.1395	0.0558
	mesaconate (methylfumarate)	GC/MS	638129	0.63	0.64	0.71	0.66	0.0006	0.0007	0.0003	0.0004	0.0391	0.0357	0.0002	0.0002
Oxidative Phosphorylation	pyrophosphate (PPi)	GC/MS	644102	1.55	1.21	1.76	1.47	0.9856	0.2737	0.3732	0.1628	0.9121	0.3653	0.674	0.1957
Fatty Acid, Dicarboxylate	2-hydroxyglutarate	GC/MS	43	0.96	0.9	0.87	0.91	0.2317	0.0906	0.619	0.2327	0.1107	0.0795	0.2381	0.0872
	4-hydroxy-2-oxoglutaric acid	GC/MS	599	2.68	1.82	2.16	2.17	0.0009	0.001	0.0053	0.005	0.0412	0.0373	0.0009	0.0008
	adipate	GC/MS	196	4.53	5.02	4	4.48	0.0034	0.0029	2.50E-05	0.0001	0.0104	0.0126	0.0001	0.0001
	2-hydroxyadipate	GC/MS	193530	0.77	0.86	0.78	0.8	0.0066	0.0052	0.0406	0.0275	0.0132	0.0151	0.005	0.0033
	pimelate (heptanedioate)	GC/MS	385	0.51	0.55	0.62	0.56	0.0001	0.0002	5.83E-07	1.88E-06	0.001	0.002	7.78E-07	1.50E-06
	suberate (octanedioate)	LC/MS pos	10457	1.24	1.1	1.07	1.13	0.3312	0.1178	0.976	0.3192	0.7696	0.328	0.7761	0.2185
	2-octenedioate	LC/MS neg	5354607	0.76	0.61	0.93	0.74	0.0186	0.0123	0.0105	0.0089	0.3569	0.1857	0.0142	0.0083
	azelate (nonanedioate)	LC/MS pos	2266	0.8	0.59	0.61	0.66	0.2403	0.0933	0.0001	0.0001	0.0018	0.0031	0.0009	0.0007
	sebacate (decanedioate)	LC/MS neg	5192	4.08	4.11	3.65	3.93	0.0166	0.0113	0.0637	0.0391	0.3805	0.1951	0.0331	0.0174
	3-carboxy-4-methyl-5-propyl-2-furanpropanoate (CMPF)	LC/MS neg	123979	1.49	1.73	1.25	1.46	0.0251	0.0156	0.1569	0.083	0.3549	0.1857	0.0646	0.0294
Fatty Acid Metabolism (also BCAA Metabolism)	butyrylcarnitine	LC/MS pos	439829	0.63	0.54	0.68	0.61	0.0625	0.0325	0.0448	0.0297	0.0908	0.0678	0.0254	0.0138
	propionylcarnitine	LC/MS pos	107738	1.1	1.03	1.02	1.05	0.7875	0.2311	0.8466	0.29	0.812	0.3406	0.9378	0.252
Fatty Acid Metabolism(Acyl Glycine)	hexanoylglycine	LC/MS neg	99463	1.62	1.88	1.71	1.73	0.0002	0.0003	0.0003	0.0005	0.0205	0.0215	0.0001	0.0001
Fatty Acid Metabolism(Acyl Carnitine)	acetylcarnitine	LC/MS pos	1	2.39	2.63	2.45	2.49	3.91E-05	0.0001	1.28E-06	3.79E-06	0.0002	0.0005	3.13E-07	6.70E-07
	hexanoylcarnitine	LC/MS pos	6426853	1.21	1.57	1.33	1.35	0.0587	0.0313	0.0026	0.0028	0.0722	0.057	0.0056	0.0037
	octanoylcarnitine	LC/MS pos	123701	1.06	1.4	1.3	1.23	0.2468	0.0956	0.0049	0.0047	0.0277	0.0277	0.0126	0.0074
	decanoylcarnitine	LC/MS pos	10245190	1.05	1.37	1.25	1.21	0.2596	0.0984	0.0026	0.0028	0.0312	0.0304	0.0102	0.0062
Carnitine Metabolism	carnitine	LC/MS pos	10917	1.77	1.53	1.13	1.43	0.0002	0.0002	0.0006	0.0008	0.0208	0.0217	0.0001	0.0001
	3-dehydrocarnitine*	LC/MS pos	6991982	1.07	1.16	1.01	1.08	0.3032	0.1105	0.1957	0.0979	0.4421	0.2171	0.202	0.0762
Ketone Bodies	3-hydroxybutyrate (BHBA)	GC/MS	441	18.95	24.27	19.58	20.68	1.47E-07	1.01E-06	1.59E-09	1.53E-08	2.68E-05	0.0001	2.97E-10	1.95E-09
Fatty Acid, Monohydroxy	3-hydroxysebacate	LC/MS neg	3017884	2.04	2.31	2.06	2.13	0.0094	0.0069	0.002	0.0022	0.0379	0.0349	0.0015	0.0012
	5-hydroxyhexanoate	LC/MS pos	170748	0.65	0.71	0.7	0.69	0.0057	0.0045	0.0107	0.009	0.0094	0.0117	0.0018	0.0014
Eicosanoid	prostaglandin E2	LC/MS neg	5280360	1.37	1.41	1.28	1.35	0.0174	0.0117	0.0009	0.001	0.1938	0.1231	0.0003	0.0003
Inositol Metabolism	myo-inositol	GC/MS	892	1.13	0.86	1.31	1.07	0.708	0.2141	0.6291	0.2355	0.7391	0.3193	0.8506	0.2337
	chiro-inositol	GC/MS		0.94	0.93	0.81	0.89	0.447	0.1472	0.074	0.0441	0.3952	0.2001	0.4036	0.131
	pinitol	GC/MS	164619	0.82	1.12	0.43	0.68	0.0955	0.0448	0.1098	0.0616	0.0466	0.0415	0.0451	0.022
	scyllo-inositol	GC/MS	892	0.79	0.75	0.92	0.82	0.0021	0.002	0.0049	0.0046	0.026	0.0262	0.0011	0.0009
	choline	LC/MS pos	305	1.4	1.09	1.18	1.21	0.0003	0.0004	0.0435	0.0291	0.1887	0.1208	0.0048	0.0033

Sub Pathway	Biochemical Name	Platform	PUBCHEM	Fold Difference				Statistical Values							
								(BCA)/(Normal)		(BCA)/(History)		(BCA)/(Hematuria)		(BCA)/(All Ctrls)	
				BCa / Normal	BCa / History	BCa / Hemturia	BCa / All CTRLs	p-value	q-value	p-value	q-value	p-value	q-value	p-value	q-value
Phospholipid Metabolism	choline phosphate	LC/MS pos	1014	6.35	4.99	5.85	5.67	0.0035	0.003	0.0495	0.032	0.0123	0.0144	0.0041	0.0029
	ethanolamine	GC/MS	700	0.65	0.71	0.65	0.67	0.0001	0.0001	0.0018	0.002	0.0004	0.0009	3.45E-05	4.56E-05
	phosphoethanolamine	GC/MS	1015	0.84	0.85	0.92	0.87	0.0197	0.0128	0.078	0.0458	0.1277	0.0893	0.0262	0.0142
Glycerolipid Metabolism	glycerol	GC/MS	753	3.83	2.62	3.94	3.34	0.0347	0.0205	0.0456	0.0301	0.0155	0.0172	0.0114	0.0068
	glycerol 3-phosphate (G3P)	GC/MS	754	1.02	0.9	0.97	0.96	0.3348	0.1187	0.0378	0.0261	0.4361	0.2152	0.1002	0.0422
Sphingolipid Metabolism	palmitoyl sphingomyelin	GC/MS	9939941	10.24	8.03	8	8.64	0.0032	0.0029	0.0001	0.0002	0.0112	0.0133	0.0001	0.0001
Mevalonate Metabolism	3-hydroxy-3-methylglutarate	GC/MS	1662	0.87	0.92	0.77	0.85	0.0724	0.0362	0.1474	0.0791	0.2061	0.129	0.0721	0.0322
Steroid	21-hydroxypregnenolone disulfate	LC/MS neg	134595	0.79	0.93	0.76	0.82	0.5055	0.1625	0.2463	0.1174	0.0163	0.0179	0.1256	0.0506
	5alpha-pregnan-3beta,20alpha-diol disulfate	LC/MS neg		0.88	0.91	0.59	0.76	0.6286	0.1943	0.8305	0.287	0.4291	0.2122	0.7524	0.2133
	5alpha-pregnan-3alpha,20beta-diol disulfate 1*	LC/MS neg		0.95	0.97	0.78	0.89	0.2291	0.0898	0.8799	0.2967	0.2491	0.1488	0.3757	0.1242
	pregnen-diol disulfate*	LC/MS neg		1.03	1.26	1.03	1.09	0.0611	0.032	0.2844	0.1313	0.7091	0.3095	0.2421	0.0883
	pregnanediol-3-glucuronide	LC/MS neg	123796	0.9	0.82	0.62	0.76	0.7736	0.2287	0.8736	0.2955	0.1285	0.0894	0.6389	0.1874
	cortisol	LC/MS pos	5754	2.6	1.94	1.7	2.01	5.01E-07	2.51E-06	0.0001	0.0001	0.0181	0.0196	2.71E-06	4.75E-06
	cortisone	LC/MS pos	222786	1.34	1.4	1.21	1.31	0.0001	0.0001	0.0004	0.0005	0.0388	0.0357	0.0001	0.0001
	tetrahydrocortisone	LC/MS neg	5866	1.09	1.13	1.13	1.12	0.7761	0.2287	0.6335	0.2369	0.5822	0.2677	0.6125	0.1834
	dehydroisoandrosterone sulfate (DHEA-S)	LC/MS neg	12594	1.25	0.77	0.63	0.81	0.2532	0.0964	0.0696	0.0419	0.067	0.054	0.0597	0.0277
	androsterone sulfate	LC/MS neg	159663	0.83	0.8	0.67	0.76	0.9437	0.2653	0.533	0.2106	0.0136	0.0155	0.2953	0.1039
	4-androsten-3beta,17beta-diol disulfate (1)*	LC/MS neg	10634	0.96	0.74	0.83	0.83	0.9207	0.2613	0.702	0.2535	0.1724	0.1129	0.5336	0.1643
	4-androsten-3beta,17beta-diol disulfate (2)*	LC/MS neg	10634	0.81	0.96	0.75	0.83	0.4681	0.1524	0.5578	0.2155	0.0323	0.0311	0.2324	0.0854
	5alpha-androstan-3beta,17alpha-diol disulfate	LC/MS neg		0.88	1.15	0.97	0.99	0.0889	0.0425	0.8386	0.2892	0.1753	0.1143	0.3141	0.1086
	5alpha-androstan-3beta,17beta-diol disulfate	LC/MS neg	242332	0.88	0.65	0.83	0.77	0.3798	0.1317	0.2258	0.1095	0.1205	0.0853	0.1648	0.0643
	andro steroid monosulfate (1)*	LC/MS neg		1.02	1.1	0.99	1.04	0.9322	0.2631	0.9943	0.3234	0.507	0.2389	0.8731	0.238
	andro steroid monosulfate 2*	LC/MS neg		0.94	0.94	0.75	0.87	0.7666	0.2272	0.952	0.313	0.4388	0.216	0.9423	0.2525
Primary Bile Acid Metabolism	cholate	LC/MS neg	221493	0.78	0.84	1.15	0.9	0.2117	0.0844	0.2905	0.1335	0.386	0.1972	0.2082	0.078
	glycocholate	LC/MS pos	10140	0.82	0.72	0.92	0.82	0.1105	0.0497	0.0554	0.0349	0.4291	0.2122	0.073	0.0326
Secondary Bile Acid Metabolism	deoxycholate	LC/MS neg	222528	0.75	0.74	0.98	0.81	0.0309	0.0185	0.0487	0.0317	0.1318	0.0914	0.0278	0.0149
	glycodeoxycholate	LC/MS neg	3035026	1.11	1.01	1.34	1.14	0.3828	0.1324	0.5113	0.2046	0.8873	0.3598	0.4774	0.1509
	glycolithocholate sulfate*	LC/MS neg	72222	0.73	0.94	0.65	0.76	0.0072	0.0055	0.0301	0.0217	0.0032	0.0051	0.0028	0.002
	taurolithocholate 3-sulfate	LC/MS neg	440071	0.66	0.74	0.74	0.71	0.0125	0.0089	0.0485	0.0317	0.0257	0.0261	0.0091	0.0056
	12-dehydrocholate	LC/MS neg	94235	0.87	0.82	0.66	0.77	0.0595	0.0316	0.0753	0.0444	0.0096	0.0119	0.0221	0.0123
	glycocholenate sulfate*	LC/MS neg		0.87	1.01	0.72	0.85	0.5947	0.1864	0.7158	0.2566	0.0954	0.0704	0.3837	0.1264
	taurocholenate sulfate*	LC/MS neg		1.01	0.95	1.05	1	0.5722	0.1807	0.7832	0.2746	0.5926	0.2705	0.8107	0.2254
	7-ketodeoxycholate	LC/MS neg	188292	0.83	0.73	0.92	0.82	0.0792	0.0387	0.1655	0.0868	0.0718	0.0568	0.0619	0.0286
Purine Metabolism, (Hypo)Xanthine/Inosine containing	inosine	LC/MS neg	6021	1.45	1.41	1.17	1.33	0.1531	0.0659	0.1157	0.0646	0.6958	0.3059	0.2151	0.0804
	hypoxanthine	GC/MS	790	1.06	1.23	1.01	1.09	0.9495	0.2667	0.0103	0.0087	0.654	0.2921	0.1657	0.0645
	xanthine	LC/MS pos	1188	1.33	1.6	0.95	1.24	0.0005	0.0006	2.30E-06	6.33E-06	0.0307	0.03	1.29E-05	1.92E-05
	xanthosine	LC/MS pos	64959	0.91	1.04	0.88	0.94	0.1355	0.0593	0.9144	0.3055	0.1878	0.1204	0.3063	0.1068
	urate	LC/MS neg	1175	1.18	1.2	1.02	1.13	0.0022	0.0021	0.004	0.0039	0.396	0.2001	0.0038	0.0027
	allantoin	GC/MS	204	0.59	0.47	0.66	0.56	0.0777	0.0382	1.38E-06	4.04E-06	0.0083	0.0104	0.0001	0.0001
Purine Metabolism, Adenine containing	adenosine 5'-monophosphate (AMP)	LC/MS pos	6083	20.94	11.4	9.89	12.68	1.66E-10	7.31E-09	7.22E-08	3.51E-07	0.0001	0.0003	4.60E-12	5.77E-11
	adenosine 3',5'-cyclic monophosphate (cAMP)	LC/MS neg	6076	0.79	0.96	0.81	0.85	0.0032	0.0028	0.1091	0.0613	0.0052	0.0074	0.0056	0.0037
	adenosine	LC/MS pos	60961	0.73	0.82	0.7	0.75	0.0053	0.0043	0.0343	0.0242	0.0022	0.0036	0.0024	0.0018
	adenine	GC/MS	190	0.86	1.06	0.95	0.95	0.3685	0.1282	0.702	0.2535	0.1572	0.1046	0.3404	0.1157
	N1-methyladenosine	LC/MS pos	27476	1	1.05	0.91	0.98	0.746	0.2222	0.9966	0.3235	0.2189	0.1352	0.6287	0.1855
	N6-methyladenosine	LC/MS pos	102175	1.68	1.16	0.96	1.2	0.1842	0.0765	0.6021	0.2281	0.8111	0.3405	0.4761	0.1506
	N6-carbamoylthreonyladenosine	LC/MS pos	161466	1.14	1.11	1.1	1.12	0.3403	0.1202	0.8804	0.2967	0.99	0.3852	0.6533	0.191
Purine Metabolism, Guanine containing	guanosine	LC/MS neg	6802	1.15	1.37	1.15	1.22	0.6911	0.21	0.0523	0.0333	0.9098	0.3651	0.301	0.1055
	guanine	GC/MS	764	0.94	1.25	1.19	1.11	0.2865	0.1056	0.4869	0.1969	0.6502	0.2908	0.956	0.2549
	7-methylguanine	LC/MS pos	11361	1.22	1.32	1.1	1.2	0.003	0.0027	0.0006	0.0008	0.3608	0.187	0.0017	0.0013
	N1-methylguanosine	LC/MS pos	96373	0.96	1.04	0.94	0.98	0.2086	0.0834	0.7428	0.2641	0.09	0.0673	0.2508	0.0909
	N2-methylguanosine	LC/MS pos	3035422	1.01	1.19	0.97	1.05	0.746	0.2222	0.0623	0.0386	0.4601	0.2239	0.5545	0.1692
	N2,N2-dimethylguanosine	LC/MS pos	92919	0.95	1.01	1	0.99	0.4838	0.1569	0.5714	0.2183	0.4632	0.2246	0.4404	0.1409
Pyrimidine Metabolism, Orotate containing	N-carbamoylaspartate	GC/MS	93072	0.43	0.52	0.68	0.53	0.0061	0.0048	0.0008	0.001	0.0027	0.0044	0.0003	0.0003
	orotate	GC/MS	967	1.65	1.64	1.34	1.53	0.4726	0.1537	0.7976	0.2783	0.1471	0.099	0.568	0.1722
	orotidine	LC/MS neg	92751	1.57	1.35	1.5	1.47	0.359	0.1255	0.487	0.1969	0.7695	0.328	0.4261	0.1371
	2,4-dioxo-1H-pyrimidine-5-carboxylic acid	LC/MS pos	90301	0.75	0.8	0.86	0.8	0.0519	0.0284	0.4624	0.1906	0.2085	0.1303	0.1374	0.055
Pyrimidine Metabolism, Uracil containing	uridine	LC/MS neg	6029	1.37	1.41	1.08	1.27	0.1068	0.0482	0.0719	0.043	0.9777	0.3818	0.1269	0.0511
	uracil	GC/MS	1174	0.78	0.93	0.69	0.79	0.0259	0.0161	0.445	0.1876	0.0057	0.0078	0.0397	0.0202
	pseudouridine	LC/MS neg	15047	1	1.01	0.99	1	0.7791	0.2291	0.6809	0.2491	0.445	0.2179	0.5916	0.1782
	5,6-dihydrouracil	GC/MS	649	0.97	0.94	0.86	0.92	0.7147	0.2157	0.6496	0.2414	0.262	0.1547	0.4876	0.1523
	4-ureidobutyrate	LC/MS pos	1571307	0.88	0.86	0.85	0.86	0.1731	0.0731	0.0073	0.0066	0.0073	0.0095	0.0081	0.0051
	3-ureidopropionate	LC/MS pos	111	0.93	0.96	0.87	0.92	0.8578	0.2477	0.8804	0.2967	0.0607	0.0501	0.6973	0.2004
	beta-alanine	GC/MS	239	0.64	0.62	0.7	0.65	0.8012	0.2343	0.404	0.1741	0.6847	0.302	0.6645	0.1938
	N-acetyl-beta-alanine	LC/MS pos	76406	0.94	0.99	0.86	0.93	0.2289	0.0898	0.8131	0.2823	0.2246	0.1379	0.3455	0.1171
Pyrimidine Metabolism, Cytidine containing	cytidine	LC/MS pos	6175	0.84	0.62	0.63	0.68	0.113	0.0506	0.0031	0.0032	0.0137	0.0156	0.0057	0.0037
	3-methylcytidine	LC/MS pos	159649	1	1	0.98	1	0.6654	0.2035	0.8533	0.2912	0.8944	0.3604	0.907	0.2454
	N4-acetylcytidine	LC/MS pos	107461	1.04	1.02	0.91	0.99	0.7406	0.2212	0.8691	0.2951	0.2873	0.1652	0.6035	0.1812
Pyrimidine Metabolism, Thymine containing	thymine	GC/MS	1135	0.69	0.93	0.7	0.76	0.0148	0.0102	0.6641	0.245	0.0079	0.0101	0.052	0.0248
	5,6-dihydrothymine	GC/MS	93556	0.93	0.83	1	0.92	0.324	0.1157	0.2385	0.1145	0.8178	0.3423	0.2985	0.1048
	3-aminoisobutyrate	GC/MS	64956	1.65	1.1	1.43	1.36	0.1983	0.08	0.7931	0.277	0.263	0.1552	0.336	0.1145
Nicotinate and Nicotinamide Metabolism	quinolinate	LC/MS pos	1066	0.91	0.83	0.99	0.91	0.2151	0.0857	0.1499	0.0799	0.5543	0.2583	0.1673	0.065
	nicotinate	GC/MS	938	0.84	0.78	1.1	0.89	0.3015	0.1101	0.0264	0.0194	0.9581	0.3766	0.1247	0.0504
	nicotinamide	GC/MS	936	1.28	1	1.24	1.16	0.5612	0.1779	0.504	0.2029	0.9739	0.3814	0.5664	0.1719
	trigonelline (N'-methylnicotinate)	LC/MS pos	5570	1	0.88	1.16	1	0.4681	0.1524	0.0557	0.0351	0.6979	0.3065	0.2574	0.0931
	nicotinurate	LC/MS pos	68499	9.19	9.46	8.98	9.21	0.0898	0.0428	0.2103	0.104	0.4795	0.2293	0.0984	0.0416
	N1-Methyl-2-pyridone-5-carboxamide	LC/MS pos	69698	0.91	0.9	1.03	0.94	0.0029	0.0026	0.0026	0.0027	0.7166	0.3118	0.0053	0.0035
Riboflavin Metabolism	riboflavin (Vitamin B2)	LC/MS pos	493570	0.24	0.18	0.4	0.25	0.0005	0.0006	0.0001	0.0001	0.0562	0.0475	0.0001	0.0001
Pantothenate and CoA Metabolism	pantothenate	LC/MS pos	6613	0.57	0.48	0.71	0.57	0.0013	0.0013	0.0005	0.0006	0.0529	0.046	0.0003	0.0003
Ascorbate and Aldarate Metabolism	gulono-1,4-lactone	GC/MS	439373	1.87	1.71	1.85	1.81	0.0007	0.0008	0.0045	0.0043	0.0043	0.0064	0.0003	0.0003
	ascorbate (Vitamin C)	GC/MS		0.24	0.32	0.78	0.35	0.0701	0.0356	0.1658	0.0869	0.4421	0.2171	0.1029	0.0431
	threonate	GC/MS	151152	0.69	0.69	0.94	0.76	0.035	0.0206	0.0039	0.0039	0.0792	0.0611	0.0054	0.0036
	arabonate	GC/MS	122045	0.84	0.82	0.87	0.84	0.0138	0.0096	0.003	0.0031	0.0361	0.0337	0.0022	0.0017
	oxalate (ethanedioate)	GC/MS	971	2.11	1.4	2	1.78	0.0711	0.0358	0.377	0.164	0.2473	0.1484	0.1371	0.055
Tocopherol Metabolism	alpha-tocopherol	GC/MS	14985	6.15	3.78	5.31	4.87	7.98E-06	2.28E-05	0.0005	0.0007	0.0002	0.0005	3.14E-08	9.85E-08
	gamma-CEHC	LC/MS neg	133098	0.62	0.8	0.56	0.65	0.0524	0.0286	0.6516	0.2419	0.0289	0.0286	0.1088	0.0451
	alpha-CEHC glucuronide*	LC/MS neg		0.46	0.31	0.72	0.44	1.19E-06	5.01E-06	1.56E-07	6.67E-07	0.0001	0.0004	1.77E-08	6.24E-08

Sub Pathway	Biochemical Name	Platform	PUBCHEM	Fold Difference: BCa / Normal	Fold Difference: BCa / History	Fold Difference: BCa / Hemturia	Fold Difference: BCa / All CTRLs	Statistical Values: (BCA)/(Normal) p-value	(BCA)/(Normal) q-value	(BCA)/(History) p-value	(BCA)/(History) q-value	(BCA)/(Hematuria) p-value	(BCA)/(Hematuria) q-value	(BCA)/(All Ctrls) p-value	(BCA)/(All Ctrls) q-value
Biotin Metabolism	biotin	LC/MS pos	171548	0.5	0.46	0.74	0.54	0.002	0.0019	0.0005	0.0007	0.0096	0.0119	0.0003	0.0003
Folate Metabolism	5-methyltetrahydrofolate (5MeTHF)	LC/MS neg	146	0.45	0.47	0.5	0.47	0.003	0.0027	0.0097	0.0084	0.1391	0.0944	0.0068	0.0044
Tetrahydrobiopterin Metabolism	dihydrobiopterin	LC/MS pos	1879	0.91	1.22	0.97	1.01	0.1908	0.0781	0.1912	0.0963	0.3568	0.1857	0.898	0.2438
Pterin Metabolism	neopterin	LC/MS pos	4455	1.17	1.04	1.14	1.12	0.0284	0.0174	0.2755	0.1279	0.1285	0.0894	0.0645	0.0294
	7,8-dihydroneopterin	GC/MS	65074	0.88	1.05	1.08	1	0.5879	0.1844	0.5352	0.2106	0.452	0.2207	0.7567	0.2143
Hemoglobin and Porphyrin Metabolism	bilirubin (Z,Z)	LC/MS neg	5280352	1.03	0.93	1.16	1.03	0.8952	0.256	0.7727	0.2726	0.2311	0.1411	0.6923	0.1994
	bilirubin (E,E)*	LC/MS pos	5315454	1.16	1.31	1.19	1.22	0.8829	0.2539	0.0655	0.0401	0.4184	0.2079	0.238	0.0872
	biliverdin	LC/MS neg	5353439	1.2	1.17	1.18	1.19	0.0087	0.0065	0.0434	0.029	0.1264	0.0886	0.0021	0.0016
	I-urobilinogen	LC/MS neg	26818	0.63	0.67	0.48	0.58	0.054	0.0294	0.5187	0.2071	0.0617	0.0506	0.1133	0.0467
	L-urobilin	LC/MS pos	5280818	0.76	0.78	0.62	0.71	3.60E-05	0.0001	0.0035	0.0035	0.0007	0.0014	0.0001	0.0001
Vitamin B6 Metabolism	pyridoxine (Vitamin B6)	LC/MS pos	1054	0.43	0.77	0.85	0.62	0.0204	0.0131	0.0457	0.0301	0.1328	0.0914	0.0417	0.021
	pyridoxal	LC/MS pos	1050	0.41	0.34	0.48	0.4	0.0022	0.0021	0.0001	0.0001	0.0008	0.0017	3.42E-05	4.54E-05
	pyridoxate	LC/MS neg	6723	0.33	0.3	0.5	0.35	2.10E-05	4.74E-05	5.41E-07	1.79E-06	0.0002	0.0005	1.48E-07	3.49E-07
Benzoate Metabolism	hippurate	LC/MS pos	464	0.97	0.83	0.9	0.9	0.3205	0.1147	0.0137	0.0111	0.0726	0.0571	0.0323	0.017
	2-methylhippurate	LC/MS neg	91637	2.9	2.51	2.24	2.52	6.20E-07	2.86E-06	0.0051	0.0049	0.0063	0.0084	4.56E-06	7.70E-06
	2-hydroxyhippurate (salicylurate)	LC/MS neg	10253	0.13	0.17	0.21	0.16	8.68E-10	2.37E-08	2.95E-08	1.68E-07	0.001	0.0019	8.11E-10	4.69E-09
	3-hydroxyhippurate	LC/MS neg	450268	0.4	0.31	0.53	0.39	1.69E-07	1.10E-06	9.33E-11	1.27E-09	2.15E-05	0.0001	6.76E-11	5.42E-10
	4-hydroxyhippurate	LC/MS neg	151012	0.77	0.52	0.6	0.61	0.0011	0.0012	3.05E-07	1.10E-06	1.94E-06	1.66E-05	1.86E-07	4.30E-07
	mandelate	GC/MS	1292	0.78	0.76	0.79	0.78	0.0008	0.0009	1.45E-05	3.09E-05	0.0047	0.0067	1.69E-05	2.39E-05
	3-hydroxymandelate	GC/MS	86957	0.22	1.04	0.28	0.33	0.1023	0.0467	0.2566	0.1212	0.692	0.3045	0.1865	0.0712
	4-hydroxymandelate	GC/MS	328	0.72	0.71	0.68	0.7	0.0002	0.0003	0.0006	0.0008	0.0003	0.0006	2.38E-05	3.21E-05
	benzoate	GC/MS	243	1.01	0.83	1.06	0.96	0.0663	0.0341	0.8917	0.2994	0.7016	0.3075	0.4421	0.1413
	4-hydroxybenzoate	GC/MS	135	0.77	0.55	0.74	0.67	0.0188	0.0123	0.0002	0.0003	0.0327	0.0313	0.0007	0.0006
	3-hydroxybenzoate	GC/MS	7420	0.79	0.66	0.84	0.76	0.1883	0.0772	0.0043	0.0042	0.7809	0.3315	0.0424	0.0212
	2,4,6-trihydroxybenzoate	LC/MS neg	66520	0.37	0.86	0.6	0.55	0.0006	0.0007	0.1562	0.0828	0.007	0.0092	0.0068	0.0044
	catechol sulfate	LC/MS neg	3083879	0.65	0.62	0.63	0.63	1.88E-05	4.47E-05	1.59E-07	6.70E-07	0.0001	0.0003	5.68E-08	1.59E-07
	p-hydroxybenzaldehyde	GC/MS	126	0.98	1.06	1.17	1.06	0.3994	0.1353	0.5333	0.2106	0.9147	0.3653	0.5388	0.1653
	methyl-4-hydroxybenzoate	GC/MS	7456	7.51	0.44	8.88	1.2	0.0001	0.0002	2.42E-06	6.55E-06	5.46E-06	3.34E-05	4.39E-09	1.81E-08
	2-ethylphenylsulfate	LC/MS neg		1.96	1.03	1.03	1.22	0.1633	0.0699	0.3278	0.1472	0.9288	0.3686	0.9563	0.2549
	3-ethylphenylsulfate*	LC/MS neg		1.17	0.99	1.14	1.09	0.6486	0.1991	0.0521	0.0332	0.9574	0.3766	0.2502	0.0908
	4-ethylphenylsulfate	LC/MS neg		0.8	1.25	0.83	0.92	0.1795	0.0751	0.0993	0.0568	0.2414	0.1461	0.0909	0.0392
	4-vinylphenol sulfate	LC/MS neg	6426766	0.76	0.67	0.69	0.71	0.0043	0.0036	0.0111	0.0093	0.0628	0.0512	0.0033	0.0024
Xanthine Metabolism	caffeine	LC/MS pos	2519	0.68	1.09	0.63	0.75	0.1932	0.0787	0.0539	0.0341	0.1084	0.0783	0.0525	0.0249
	paraxanthine	LC/MS pos	4687	1.3	1.16	1.23	1.23	0.775	0.2287	0.6029	0.2281	0.3528	0.1857	0.9053	0.2454
	theobromine	LC/MS pos	5429	0.64	0.75	0.87	0.74	0.0238	0.0149	0.2006	0.0999	0.8687	0.3563	0.1134	0.0467
	1-methylurate	LC/MS pos	69726	1.2	1	1.25	1.14	0.9553	0.2673	0.1748	0.0908	0.6504	0.2908	0.5995	0.1803
	1,3-dimethylurate	LC/MS pos	70346	1.03	1.06	1.27	1.11	0.6058	0.1889	0.7045	0.254	0.4662	0.2255	0.852	0.2337
	1,7-dimethylurate	LC/MS neg	91611	0.83	0.87	1.06	0.91	0.0486	0.0268	0.0631	0.0388	0.8746	0.3568	0.0959	0.0409
	1,3,7-trimethylurate	LC/MS neg	79437	0.67	0.8	0.8	0.75	0.0318	0.019	0.0179	0.0139	0.2597	0.1538	0.0184	0.0105
	1-methylxanthine	LC/MS pos	80220	1.11	1.13	1.22	1.15	0.1219	0.0541	0.0832	0.0485	0.8944	0.3604	0.1497	0.0588
	3-methylxanthine	LC/MS pos	70639	0.62	0.78	0.87	0.74	0.016	0.011	0.0429	0.0288	0.6831	0.3016	0.0372	0.019
	7-methylxanthine	LC/MS pos	68374	0.68	0.78	0.93	0.79	0.0333	0.0198	0.1807	0.0927	0.8217	0.3436	0.1147	0.0471
	5-acetylamino-6-amino-3-methyluracil	LC/MS neg	88299	0.49	0.4	1.01	0.54	0.0175	0.0118	0.0006	0.0007	0.5853	0.2686	0.0049	0.0033
	5-acetylamino-6-formylamino-3-methyluracil	LC/MS neg	108214	0.72	0.92	1.21	0.91	0.0134	0.0094	0.1068	0.0602	0.99	0.3852	0.0747	0.0332
Tobacco Metabolite	cotinine	LC/MS pos	854019	7.66	2.24	2.57	3.1	0.0068	0.0053	0.9846	0.3213	0.965	0.379	0.3588	0.1201
	hydroxycotinine	LC/MS pos	10219774	2.92	1.28	0.93	1.36	0.0221	0.014	0.8433	0.2894	0.9761	0.3815	0.3515	0.1185
	cotinine N-oxide	LC/MS pos	9815514	2.08	1.18	1.17	1.37	0.0246	0.0153	0.8503	0.2908	0.836	0.3472	0.5464	0.167
Food Component/Plant	sulforaphane-cysteine	LC/MS pos		0.95	0.52	0.95	0.75	0.0832	0.0403	0.0933	0.0538	0.2935	0.1652	0.1109	0.0459
	sulforaphane-N-acetyl-cysteine	LC/MS neg		0.79	0.53	0.82	0.69	0.0485	0.0268	0.1559	0.0828	0.2454	0.148	0.0961	0.0409
	sucralose	LC/MS neg	71485	0.4	0.58	0.34	0.42	0.0028	0.0025	0.0488	0.0317	0.303	0.1697	0.0164	0.0094
	2,5-furandicarboxylic acid	LC/MS pos	76720	0.57	0.21	0.76	0.38	0.0014	0.0015	5.33E-07	1.78E-06	0.0012	0.0022	2.84E-06	4.94E-06
	1-kestose	GC/MS	440080	0.37	0.59	0.53	0.47	8.32E-09	1.14E-07	3.40E-08	1.87E-07	7.65E-08	1.39E-06	5.50E-11	4.68E-10
	2,3-butanediol	GC/MS	262	0.44	0.35	0.58	0.44	0.1016	0.0466	0.1743	0.0907	0.0717	0.0568	0.0654	0.0296
	levulinate (4-oxovalerate)	LC/MS pos	11579	1.21	1.44	1.25	1.29	0.7452	0.2222	0.2313	0.1118	0.9037	0.363	0.4478	0.1428
	vanillate	GC/MS	8468	0.96	0.78	1.08	0.92	0.0027	0.0025	2.03E-06	5.70E-06	0.002	0.0034	8.61E-06	1.36E-05
	1,6-anhydroglucose	GC/MS	2724705	0.68	0.45	0.74	0.59	0.0036	0.0031	0.0001	0.0002	0.0185	0.0198	0.0002	0.0002
	2,3-dihydroxyisovalerate	GC/MS	677	0.27	0.38	0.47	0.36	3.25E-06	1.13E-05	2.28E-07	8.76E-07	0.0003	0.0007	4.64E-08	1.34E-07
	2-isopropylmalate	LC/MS neg	77	0.29	0.34	0.36	0.33	8.43E-08	6.66E-07	4.80E-08	2.43E-07	1.23E-05	0.0001	1.05E-09	5.51E-09
	2-oxindole-3-acetate	LC/MS pos	3080590	0.46	0.54	0.46	0.48	6.22E-08	5.66E-07	2.98E-07	1.09E-06	6.83E-07	7.30E-06	8.99E-10	4.90E-09
	gluconate	GC/MS	10690	11.08	9.04	11.59	10.45	0.0008	0.0009	0.0044	0.0042	0.0029	0.0047	0.0003	0.0003
	abscisate	LC/MS neg	5280896	0.59	0.4	0.57	0.5	0.0353	0.0207	0.0088	0.0076	0.0036	0.0055	0.0054	0.0036
	caffeate	GC/MS	689043	0.82	0.74	0.96	0.83	0.533	0.1708	0.1212	0.0673	0.3883	0.1979	0.2064	0.0776
	3-hydroxycinnamate (m-coumarate)	LC/MS neg	637541	0.66	0.46	0.72	0.59	0.0215	0.0137	0.0007	0.0009	0.1018	0.0746	0.0027	0.002
	chlorogenate	LC/MS neg	5315832	1	0.98	1	0.99	0.3964	0.1351	0.2946	0.1346			0.3892	0.1272
	ciliatine (2-aminoethylphosphonate)	GC/MS	339	1.93	0.23	0.22	0.32	0.0006	0.0007	0.0028	0.0029	0.0066	0.0087	0.0004	0.0003
	cinnamoylglycine	LC/MS neg	709625	0.49	0.4	0.47	0.45	1.83E-06	7.14E-06	1.42E-05	3.05E-05	6.50E-06	3.78E-05	8.77E-08	2.26E-07
	citraconate	GC/MS	643798	0.89	0.97	0.94	0.93	0.554	0.1762	0.8736	0.2955	0.3138	0.1727	0.9794	0.2591
	daidzein	LC/MS neg	5281708	0.9	0.78	0.41	0.62	0.1189	0.053	0.2358	0.1136	0.5561	0.2589	0.1806	0.0696
	dihydroferulic acid	LC/MS neg	14340	0.74	0.45	0.43	0.51	0.0197	0.0128	0.0007	0.0009	0.002	0.0034	0.0006	0.0005
	enterolactone	LC/MS neg	10685477	0.81	0.52	0.83	0.69	0.4098	0.1373	0.1782	0.0921	0.6047	0.2745	0.2643	0.095
	erythritol	GC/MS	222285	1.16	1.28	1.25	1.23	0.0191	0.0125	0.0677	0.0411	0.022	0.0228	0.0125	0.0074
	fucitol	GC/MS	3429	0.89	0.86	0.96	0.9	0.4398	0.1456	0.0868	0.0504	0.3687	0.1902	0.1554	0.061
	Isobar: glucuronate, galacturonate, 5-keto-gluconate	LC/MS neg		0.89	0.89	0.98	0.92	0.8183	0.2383	0.1729	0.0901	0.285	0.1652	0.3702	0.1231
	homocitrate	GC/MS	439459	0.92	0.94	0.94	0.93	0.0928	0.0439	0.0787	0.0461	0.0561	0.0475	0.0364	0.0187
	homostachydrine*	LC/MS pos	441447	0.99	1.08	0.78	0.93	0.8607	0.2482	0.6049	0.2287	0.7166	0.3118	0.6647	0.1938
	Isobar: dihydrocaffeate, 3,4-dihydroxycinnamate	GC/MS		1.03	0.88	1.11	1	0.2959	0.1086	0.29	0.1335	0.998	0.3872	0.346	0.1172
	malitol	GC/MS	3871	1.03	1.04	1.08	1.05	0.9293	0.2625	0.9293	0.3088	0.187	0.1201	0.8406	0.2317
	methyl indole-3-acetate	LC/MS pos	74706	1.36	1.34	1.28	1.33	0.9256	0.2622	0.3917	0.1696	0.6814	0.3011	0.7408	0.2108
	N-(2-furoyl)glycine	LC/MS neg	21863	0.53	0.48	0.59	0.53	0.0006	0.0008	2.21E-07	8.62E-07	0.0005	0.0011	7.84E-07	1.50E-06
	quebrachitol	GC/MS	151108	1.99	2.38	1.67	1.98	8.03E-06	2.28E-05	5.00E-05	0.0001	0.0087	0.0108	2.75E-07	6.07E-07
	quinate	GC/MS	6508	0.82	0.64	0.95	0.78	0.4974	0.1603	0.007	0.0064	0.3058	0.1705	0.1858	0.0711
	saccharin	LC/MS neg	5143	0.51	1.41	1.36	0.88	0.9953	0.2754	0.2761	0.128	0.1725	0.1129	0.3513	0.1185
	sinapate	GC/MS	637775	0.64	0.45	0.68	0.57	0.1311	0.0579	0.008	0.007	0.0836	0.0635	0.0185	0.0105
	stachydrine	LC/MS pos	115244	1.27	0.85	1.06	1.03	0.3049	0.1106	0.8198	0.2841	0.5297	0.2488	0.4831	0.1519
	sulforaphane	LC/MS pos	5350	0.85	0.61	0.82	0.74	0.4848	0.157	0.1842	0.0932	0.5921	0.2705	0.4218	0.1359
	tartarate	GC/MS	444305	0.24	0.29	0.35	0.29	5.26E-06	1.62E-05	5.48E-07	1.80E-06	1.40E-05	0.0001	2.84E-08	9.12E-08

Sub Pathway	Biochemical Name	Platform	PUBCHEM	Fold Difference				Statistical Values							
								(BCA)/(Normal)		(BCA)/(History)		(BCA)/(Hematuria)		(BCA)/(All Ctrls)	
				BCa Normal	BCa History	BCa Hemturia	BCa All CTRLs	p-value	q-value	p-value	q-value	p-value	q-value	p-value	q-value
	thymol sulfate	LC/MS neg		0.34	0.43	0.5	0.41	0.0073	0.0055	2.94E-05	0.0001	0.0035	0.0054	0.0001	0.0001
Drug	vancomycin	LC/MS pos	14969	1.02	1.02	1.02	1.02	0.2509	0.0956	0.183	0.0927	0.3569	0.1857	0.0455	0.022
	Gentamycin*	LC/MS pos	72395	1.41	1.41	1.41	1.41	0.0437	0.0247	0.0197	0.0152	0.1036	0.0755	0.0005	0.0004
	2-hydroxyacetaminophen sulfate*	LC/MS neg		2.05	1.83	3.82	2.32	0.5545	0.1762	0.9284	0.3087	0.6295	0.2836	0.6761	0.196
	2-methoxyacetaminophen sulfate*	LC/MS neg		1.01	1.12	1.17	1.09	0.1833	0.0762	0.5281	0.2095	0.2243	0.1379	0.2318	0.0854
	3-(cystein-S-yl)acetaminophen*	LC/MS pos	5233914	1.65	1.1	1.82	1.45	0.261	0.0988	0.4877	0.197	0.4366	0.2152	0.3091	0.1075
	4-acetaminophen sulfate	LC/MS neg	83939	1.61	1.42	3.73	1.88	0.5421	0.1732	0.7818	0.2744	0.6572	0.2932	0.6118	0.1833
	4-acetamidophenol	GC/MS	1983	1.86	1.01	1.87	1.46	0.0601	0.0317	0.2257	0.1095	0.0816	0.0626	0.0644	0.0294
	p-acetamidophenylglucuronide	LC/MS neg	83944	1.49	2.44	3.72	2.22	0.2115	0.0844	0.457	0.1906	0.3227	0.176	0.2492	0.0907
	2-methoxyacetaminophen glucuronide*	LC/MS pos		1.56	1.28	1.69	1.49	0.7535	0.224	0.9313	0.3091	0.561	0.2608	0.7378	0.2103
	salicyluric glucuronide*	LC/MS neg		0.13	0.18	0.21	0.17	5.14E-10	1.69E-08	1.40E-07	6.05E-07	0.0109	0.0131	6.03E-09	2.32E-08
	ibuprofen acyl glucuronide	LC/MS neg	163959	0.19	0.08	0.03	0.06	0.0137	0.0096	0.1186	0.066	0.1359	0.0931	0.0475	0.0229
	ibuprofen	LC/MS neg	3672	0.59	0.9	0.76	0.73	0.0832	0.0403	0.4633	0.1906	0.2935	0.1652	0.2201	0.0819
	4-acetylphenol sulfate	LC/MS neg	4684006	0.59	0.57	0.55	0.57	2.25E-05	4.94E-05	2.42E-06	6.55E-06	1.24E-05	0.0001	1.29E-07	3.11E-07
	allopurinol riboside	LC/MS neg		0.73	1.59	1.41	1.11	0.9111	0.2593	0.0379	0.0261	0.4964	0.2353	0.2359	0.0866
	amitriptyline	LC/MS pos	2160												
	atenolol	LC/MS pos	2249	0.22	0.68	1.22	0.44	0.6704	0.2044	0.8171	0.2834	0.4069	0.2036	0.7914	0.222
	benzoylecgonine	LC/MS pos	442997	1	1	1	1			0.4633	0.1906			0.6237	0.1844
	celecoxib	LC/MS neg	2662	0.99	1	1	1	0.3964	0.1351					0.6237	0.1844
	citalopram	LC/MS pos	2771	1.3	1.1	1.19	1.19	0.056	0.0303	0.176	0.0911	0.4006	0.2015	0.0681	0.0307
	codeine	LC/MS pos	5284371	0.94	1.01	1.02	0.99	0.8459	0.2447	0.9373	0.3103	0.9451	0.3725	0.9973	0.2634
	desmethylnaproxen sulfate*	LC/MS neg	184679	0.75	1.2	1.59	1.07	0.0094	0.0069	0.1241	0.0684	0.1398	0.0947	0.0429	0.0214
	diphenhydramine	LC/MS pos	3100	0.73	1.17	1.03	0.94	0.613	0.1905	0.9726	0.3187	0.832	0.3458	0.8028	0.2234
	doxycycline	LC/MS pos		1	1	1	1								
	duloxetine	LC/MS pos	60835	1	1	1	1								
	erlotinib	LC/MS pos	176870	1	1	1	1								
	fluoxetine	LC/MS pos	3386	0.99	1	1	1	0.3964	0.1351	0.4633	0.1906			0.4838	0.1519
	fluvoxamine	LC/MS pos	5324346	1	1	1	1	0.3964	0.1351					0.6237	0.1844
	gabapentin	LC/MS pos	446;6919078	0.18	0.11	1.72	0.2	0.5777	0.1818	0.8259	0.2857	0.8543	0.353	0.9387	0.252
	hydrochlorothiazide	LC/MS neg	3639	0.33	2.66	0.39	0.5	0.0002	0.0003	0.3685	0.1611	0.4728	0.2274	0.0351	0.0182
	hydroquinone sulfate	LC/MS neg	161220	0.78	0.64	0.58	0.66	0.0061	0.0048	0.0007	0.0008	2.28E-05	0.0001	0.0001	0.0001
	ketamine	LC/MS pos	3821	3.69	3.69	3.69	3.69	1.36E-05	3.42E-05	5.74E-07	1.86E-06	0.0004	0.0009	1.72E-13	4.13E-12
	lidocaine	LC/MS pos	3676	43.18	0.84	1.18	1.46	1.70E-10	7.31E-09	2.17E-06	6.05E-06	0.0001	0.0002	1.43E-09	7.24E-09
	atorvastatin (lipitor)	LC/MS neg	60823	2.8	2.63	2.52	2.64	0.4448	0.1466	0.0382	0.0263	0.2895	0.1652	0.0899	0.0388
	meprobamate*	LC/MS pos	4064	0.98	0.99	0.98	0.98	0.3964	0.1351	0.4633	0.1906	0.2935	0.1652	0.3892	0.1272
	metformin	LC/MS pos	4091	0.6	0.9	0.75	0.73	0.0299	0.0181	0.1331	0.0725	0.3758	0.1932	0.0755	0.0334
	metoprolol	LC/MS pos	4171	0.19	0.45	0.42	0.3	0.5575	0.1769	0.5641	0.2172	0.2507	0.1494	0.7471	0.2122
	metoprolol acid metabolite*	LC/MS pos	62936	0.23	0.69	0.37	0.35	0.1976	0.08	0.9615	0.3153	0.2358	0.1433	0.3758	0.1242
	milnacipran	LC/MS pos		1.04	1.04	1.04	1.04	0.1014	0.0466	0.0579	0.0362	0.187	0.1201	0.0045	0.0031
	mirtazapine	LC/MS pos	4205	1	1	1	1			0.4633	0.1906			0.6237	0.1844
	naproxen	LC/MS neg	156391	1.16	1.3	1.43	1.29	0.085	0.0411	0.4751	0.1939	0.9451	0.3725	0.2742	0.0975
	nicotine	LC/MS pos	89594	3.58	1.21	0.96	1.4	0.0559	0.0303	0.409	0.1756	0.3725	0.1917	0.9485	0.2537
	norfluoxetine	LC/MS pos	4541	1	1	1	1			0.4633	0.1906			0.6237	0.1844
	ofloxacin	LC/MS pos	4583	2.71	2.24	2.44	2.45	0.0081	0.0061	0.021	0.016	0.0469	0.0417	0.0007	0.0006
	paroxetine	LC/MS pos	43815	1	1	1	1								
	phenylephrine	LC/MS pos	6041	0.98	0.99	0.94	0.97	0.3964	0.1351	0.4633	0.1906	0.0638	0.0518	0.2639	0.095
	phenylpropanolamine	LC/MS pos	26934	1	0.99	1	1	0.3964	0.1351	0.4633	0.1906	0.2935	0.1652	0.3892	0.1272
	pivaloylcarnitine	LC/MS pos	126894	4.69	1.55	6.48	2.96	0.0575	0.0309	0.1396	0.0755	0.2022	0.1272	0.0633	0.0291
	pseudoephedrine	LC/MS pos	7028	2.39	2.5	0.51	1.08	0.0129	0.0092	0.0001	0.0002	0.1954	0.1237	0.0001	0.0001
	quetiapine	LC/MS pos	5002	1	1	1	1								
	quinine	LC/MS pos	2728270	1	1	0.99	1			0.4633	0.1906	0.2935	0.1652	0.4838	0.1519
	ranitidine	LC/MS pos	3001055	0.8	0.21	1.01	0.43	0.4368	0.1449	0.8839	0.2973	0.586	0.2686	0.5902	0.178
	rosuvastatin	LC/MS neg	446157	1.01	0.92	0.94	0.95	0.777	0.2287	0.8583	0.2923	0.9023	0.3628	0.8725	0.238
	salicylate	GC/MS	338	0.11	0.09	0.2	0.12	3.29E-05	0.0001	0.0005	0.0007	0.0547	0.047	0.0001	0.0001
	sertraline	LC/MS pos	68617	1	1	1	1								
	Tetracycline*	LC/MS pos	54675776	1	1	1	1								
	topiramate	LC/MS pos	5284627	1	1	1	1								
	venlafaxine	LC/MS pos	5656	0.72	0.82	0.26	0.46	0.0204	0.0131	0.1347	0.0733	0.1328	0.0914	0.0677	0.0306
Chemical	pentaethylene glycol	LC/MS pos	62551	4.14	3.52	2.54	3.26	0.0008	0.0009	0.071	0.0426	0.0072	0.0095	0.0027	0.002
	hexaethylene glycol	LC/MS pos	17472	4.87	3.53	1.53	2.62	0.0042	0.0035	0.0878	0.0507	0.0136	0.0155	0.0071	0.0045
	heptaethylene glycol	LC/MS pos	79718	6.85	3.22	1.1	2.2	0.0064	0.005	0.1926	0.0969	0.0127	0.0148	0.0145	0.0084
	octaethylene glycol	LC/MS pos	78798	4.3	2.28	1.29	2.08	0.0764	0.0379	0.3357	0.1497	0.0466	0.0415	0.079	0.0349
	diglycerol	LC/MS pos	42953	0.92	0.77	0.76	0.81	0.3053	0.1106	0.0171	0.0135	0.0379	0.0349	0.0287	0.0153
	1,2-propanediol	GC/MS	1030	5.37	3.11	5.95	4.43	1.23E-05	3.19E-05	0.0015	0.0017	0.0159	0.0175	0.0001	0.0001
	3-hydroxypyridine	GC/MS	7971	0.72	0.72	0.79	0.74	0.0373	0.0217	0.0043	0.0042	0.1669	0.1102	0.0081	0.005
	2-pyrrolidinone	GC/MS	12025	0.57	0.71	0.66	0.64	0.0007	0.0008	0.0071	0.0064	0.0061	0.0083	0.0005	0.0005
	1-(3-aminopropyl)-2-pyrrolidone	LC/MS pos	82111	1.6	1.23	1.37	1.38	0.0033	0.0029	0.1524	0.081	0.096	0.0707	0.0184	0.0105
	2-oxo-1-pyrrolidinepropionate	LC/MS pos	3146688	1.09	0.93	1.07	1.02	0.3057	0.1106	0.0439	0.0293	0.2883	0.1652	0.0858	0.0373
	ethyl glucuronide	LC/MS pos	152226	0.24	0.21	0.57	0.28	0.0774	0.0382	0.6125	0.2307	0.8971	0.3611	0.3538	0.119
	phenolphthalein beta-D-glucuronide	LC/MS pos	3032634	1	1	1	1								
	bisphenol A monosulfate	LC/MS neg		0.38	0.6	0.59	0.5	0.0002	0.0003	0.0067	0.0061	0.0077	0.0099	0.0009	0.0008
	2-hydroxyisobutyrate	GC/MS	11671	1.08	1.24	1.15	1.15	0.6445	0.1981	0.0677	0.0411	0.4016	0.2015	0.1811	0.0697
	cyclohexylamine*	GC/MS	7965	1.24	0.83	0.87	0.95	0.3132	0.1125	0.0617	0.0384	0.19	0.1212	0.3632	0.1213
	ectoine	LC/MS pos	126041	0.73	0.75	0.67	0.71	0.0653	0.0337	0.1387	0.0751	0.1076	0.078	0.0535	0.0252
	glycolate (hydroxyacetate)	GC/MS	757	0.87	0.88	0.72	0.82	0.0637	0.033	0.1038	0.0589	0.0166	0.018	0.0255	0.0138
	trizma acetate	GC/MS	6503	1.05	1.05	0.93	1	0.1014	0.0466	0.0579	0.0362	0.3157	0.1733	0.4107	0.1329
	benzophenone	GC/MS	3102	1	1	1	1								
