# Supplementary material for: Bladder Cancer Biomarker Discovery Using Global Metabolomic Profiling of Urine
Source: PLoS One. 2014 Dec 26;9(12):e115870. doi: 10.1371/journal.pone.0115870 (PMC4277370; doi:10.1371/journal.pone.0115870)
Supplement: S2 Table — Cohort 2 heatmap of all measured named metabolites. Relative metabolite ratios and Wilcoxon statistical significance determinations as described for S1 Table. (PDF) [file pone.0115870.s002.pdf]

Supplementary Table S2 Cohort 2 heatmap of all measured named metabolites. Relative metabolite ratios are presented for Bca positive versus all control groups and also the combined control data set. Metabolites are grouped by their associated metabolism sub pathway. Cell colors represent: dark red = higher in BCa at p $\leq$ 0.05 significance; dark green = lower in BCa at p < 0.05; light red = higher in BCa at p $\leq$ 0.1; light green = lower in BCa at p < 0.1. The platform column designates the chromatography/MS platform the measurements were derived from. Pubchem reference numbers for the individual metabolites are given.

Sub Pathway	Biochemical Name	Platform	PUBCHEM	Fold of Change	Statistics	
				BCa / All CTRLs	BCA / All Ctrls	
					p-value	q-value
Glycine, Serine and Threonine Metabolism	glycine	GC/MS	5257127;750	1.25	0.8843	0.7128
	N-acetylglycine	203	10972	1.22	0.3638	0.6021
	sarcosine (N-Methylglycine)	GC/MS	1088;7311726	1.15	0.2857	0.5528
	dimethylglycine	GC/MS	6971056;673	1.13	0.9172	0.7194
	betaine	202	247	1	0.6226	0.6494
	serine	GC/MS	5951;6857581	1.33	0.2277	0.5494
	N-acetylserine	202	65249	1.06	0.5102	0.635
	beta-hydroxypyruvate	GC/MS	964	1.21	0.263	0.5528
	threonine	GC/MS	6971019;6288	1.43	0.3494	0.5962
	N-acetylthreonine	203	4651717	1.16	0.0989	0.4348
	allo-threonine	GC/MS	99289	1.22	0.1221	0.4441
	homoserine	GC/MS	12647;6971022	1.01	0.6978	0.6599
Alanine and Aspartate Metabolism	alanine	GC/MS	5950	1.17	0.2585	0.5528
	N-acetylalanine	203	88064	1.17	0.6623	0.6549
	aspartate	GC/MS	5960	1.91	0.0021	0.0797
	asparagine	GC/MS	6267	1.15	0.1925	0.5128
	N-acetylasparagine	202	99715	0.98	0.4334	0.63
	N-acetylaspartate (NAA)	202	65065	0.97	0.4457	0.635
Glutamate Metabolism	glutamate	GC/MS	611	2.41	3.60E-05	0.0073
	glutamine	202	5961	1.23	0.1436	0.4592
	N-acetylglutamate	202	1549099	0.97	0.4173	0.6233
	N-acetylglutamine	202	182230	1.1	0.5839	0.6421
	N-acetyl-aspartyl-glutamate (NAAG)	202	5255	1.01	0.857	0.7043
	gamma-aminobutyrate (GABA)	GC/MS	6992099;119	0.78	0.0536	0.3801
	N-methylglutamate	GC/MS	439377	1.31	0.3712	0.6032
	pyroglutamine*	202	134508	1.2	0.7869	0.686
	citramalate	GC/MS	1081	0.98	0.8137	0.6938
Histidine Metabolism	histidine	203	773;3651426	1.26	0.4925	0.635
	N-acetylhistidine	202	75619	1.18	0.8843	0.7128
	1-methylhistidine	202	92105;7020397	0.84	0.9503	0.7236
	3-methylhistidine	203	64969;6971061	0.78	0.2826	0.5528
	N-acetyl-3-methylhistidine*	202		1.1	0.8952	0.7163
	N-acetyl-1-methylhistidine*	202		1.03	0.9558	0.7251
	hydantoin-5-propionic acid	203	782	1.26	0.1361	0.4496
	trans-urocanate	202	736715	1.01	0.7762	0.6834
	cis-urocanate	203	1549103	0.9	0.4969	0.635
	formiminoglutamate	202	439233	1.17	0.3284	0.5788
	imidazole propionate	202	70630	1.67	0.5697	0.6377
	imidazole lactate	202	440129	1.03	0.2857	0.5528
	1-methylimidazoleacetate	202	75810	1.17	0.0819	0.4348
	4-imidazoleacetate	202	96215	0.92	0.2527	0.5528
	lysine	202	5962	1.42	0.4055	0.6193
	N2-acetyllysine	202	6992697;92907	0.95	0.9503	0.7236
	N6-acetyllysine	202	6991978;92832	1.02	0.9613	0.7259
	N-6-trimethyllysine	GC/MS	159659	1.7	0.0097	0.1971
	2-aminoadipate	202	469	1.05	0.8029	0.6901

Sub Pathway	Biochemical Name	Platform	PUBCHEM	Fold of Change	Statistics	
				BCa All CTRLs	BCA All Ctrls	
					p-value	q-value
Lysine Metabolism	glutarate (pentanedioate)	GC/MS	4418048	1.02	0.1513	0.4778
	glutarylcarnitine (C5)	202		1.03	0.5418	0.6359
	3-hydroxyglutarate	GC/MS	181976	0.93	0.5511	0.6359
	pipecolate	202	849	0.94	0.8679	0.7059
	N-acetyl-cadaverine	202		0.9	0.9779	0.729
	5-aminovalerate	GC/MS	6992101;138	0.92	0.9723	0.7289
	5-(galactosylhydroxy)-L-lysine	202		1.1	0.1379	0.4502
Phenylalanine and Tyrosine Metabolism	phenylalanine	202	6925665;6140	1.19	0.1742	0.4933
	N-acetylphenylalanine	203	74839	1.19	0.1787	0.5006
	4-hydroxyphenylacetate	GC/MS	4693933	**1.39**	0.0819	0.4348
	3-hydroxyphenylacetate	GC/MS	6950813	1.05	0.5282	0.6359
	phenylacetylglycine	202	68144	0.81	0.4016	0.6193
	phenylacetylglutamine	202	306137	**1.12**	0.0843	0.4348
	tyrosine	202	6057;6942100	1.21	0.4667	0.635
	N-acetyltyrosine	203	68310	1.04	0.6226	0.6494
	tyramine	202	5610	**0.89**	0.0813	0.4348
	4-hydroxyphenylpyruvate	203	979	2.6	0.1119	0.4348
	3-(4-hydroxyphenyl)lactate	GC/MS	9378	1.4	0.5373	0.6359
	phenol sulfate	203	74426	1.08	0.6825	0.6563
	p-cresol sulfate	203	4615422	0.95	0.3939	0.6193
	o-cresol sulfate	203	11615528	1.67	0.1058	0.4348
	dihydoxyphenylalanine (L-DOPA)	203	6971033;6047	1		
	dopamine	GC/MS	681	1.14	0.4499	0.635
	vanillylmandelate (VMA)	GC/MS	1245	1.05	0.4094	0.6195
	3-methoxytyrosine	202	9307	0.85	0.1361	0.4496
	3-methoxytyramine	202	1669	**0.71**	0.0817	0.4348
	3-methoxytyramine sulfate	203		1.26	0.3147	0.5749
	3,4-dihydroxyphenylacetate	GC/MS	547	0.83	0.2644	0.5528
	vanilpyruvate	203	14124	1.15	0.5124	0.635
	homovanillate (HVA)	203	6950168	0.87	0.9447	0.7236
	homovanillate sulfate	203		0.95	0.9007	0.7163
	3-methoxy-4-hydroxyphenylglycol	GC/MS	10805	1.1	0.4457	0.635
	gentisate	GC/MS	3469	1.25	0.6979	0.6599
	3-[3-(sulfooxy)phenyl]propanoic acid	203		1.22	0.6374	0.6523
	2-pentanamido-3-phenylpropanoic acid	203		1.12	0.8461	0.703
	3-(3-hydroxyphenyl)propionate	203	91	1.46	0.1972	0.5221
	5-hydroxymethyl-2-furoic acid	GC/MS	80642	0.78	0.6275	0.6495
	2-hydroxyphenylacetate	GC/MS	11970	1.09	0.172	0.4891
Tryptophan Metabolism	tryptophan	202	6923516;6305	1.3	0.39	0.6193
	N-acetyltryptophan	203	700653	**1.26**	0.0908	0.4348
	tryptamine	202	1150	1.15	0.9072	0.7164
	indolelactate	202	92904	1.17	0.9392	0.7227
	indoleacetate	202	802	1.03	0.8461	0.703
	3-indoxyl sulfate	203	10258	0.96	0.5418	0.6359
	kynurenine	202	161166;6971029	1.23	0.5604	0.6359
	kynurenate	203	3845	0.95	0.9613	0.7259
	anthranilate	202	227	1.01	0.9696	0.7281
	3-hydroxykynurenine	202	89	1.44	0.1048	0.4348
	3-hydroxyanthranilate	202	86	1.29	0.6523	0.6543
	xanthurenate	202	5699	0.84	0.1656	0.4823
	picolinate	202	1018	1.12	0.4582	0.635
	5-hydroxyindoleacetate	202	1826	1.08	0.703	0.6599
	indoleacetylglutamine	203		0.54	0.2952	0.5579
	tryptophan betaine	202	442106	1.19	0.4314	0.63

Sub Pathway	Biochemical Name	Platform	PUBCHEM	Fold of Change	Statistics	
				BCa / All CTRLs	BCA / All Ctrls	
					p-value	q-value
	C-glycosyltryptophan*	203		1.06	0.6623	0.6549
Leucine, Isoleucine and Valine Metabolism	leucine	202	7045798;6106	1.56	0.0202	0.2452
	N-acetylleucine	202	70912	1.08	0.4437	0.635
	4-methyl-2-oxopentanoate	203	70	2.59	0.3625	0.6012
	isovalerylglycine	203	546304	0.81	0.0819	0.4348
	isovalerylcarnitine	202	6426851	1.33	0.5697	0.6377
	3-methylcrotonylglycine	202	169485	0.88	0.2147	0.5317
	beta-hydroxyisovalerate	203	69362	0.86	0.7133	0.6624
	hydroxyisovaleroyl carnitine	202		1.05	0.2704	0.5528
	3-methylglutarylcarnitine (C6)	202	128145	1.23	0.3602	0.5985
	3-methylglutaconate	GC/MS	1551553	1.18	0.2021	0.5244
	3-methylglutarate	GC/MS	12284	1.33	0.0908	0.4348
	alpha-hydroxyisovalerate	GC/MS	99823	1.21	0.5164	0.635
	isoleucine	202	791	1.49	0.03	0.2939
	N-acetylisoleucine	202	2802421	0.97	0.3017	0.5635
	3-methyl-2-oxovalerate	203	47	1.07	0.2729	0.5528
	2-methylbutyrylcarnitine (C5)	202	6426901	1.14	0.4375	0.6326
	2-methylbutyrylglycine	202	193872	0.73	0.0131	0.2134
	tiglyl carnitine	202		1.15	0.5146	0.635
	tigloylglycine	202	6441567	0.92	0.4253	0.625
	3-hydroxy-2-ethylpropionate	GC/MS	188979	1.07	0.6876	0.6566
	valine	202	6971018;6287	1.38	0.0144	0.2134
	N-acetylvaline	203	66789	0.97	0.2442	0.5528
	isobutyrylcarnitine	202		1.12	0.1809	0.5022
	isobutyrylglycine	202	10855600	0.93	0.608	0.6475
	3-hydroxyisobutyrate	GC/MS	11966314	0.95	0.6523	0.6543
Methionine, Cysteine, SAM and Taurine Metabolism	methionine	GC/MS	6992087;6137	1.55	0.0743	0.4282
	N-acetylmethionine	203	448580	1.22	0.846	0.703
	N-formylmethionine	203	439750	1.07	0.9062	0.7163
	S-adenosylmethionine (SAM)	202		1.05	0.4133	0.6197
	S-adenosylhomocysteine (SAH)	202		1.27	0.129	0.4441
	cystathionine	GC/MS	439258	1.27	0.2296	0.5494
	2-aminobutyrate	GC/MS	439691;6971251	1.24	0.3861	0.6165
	2-hydroxybutyrate (AHB)	GC/MS	440864	1.5	0.2489	0.5528
	cysteine	GC/MS	5862	1.3	0.021	0.247
	cystine	GC/MS	67678	1.58	0.1492	0.4736
	S-methylcysteine	GC/MS	7058174;24417	1.31	0.2469	0.5528
	S-methylcysteine sulfoxide	203	82142	0.84	0.857	0.7043
	taurine	203	1123;4068592	1.05	0.1048	0.4348
Urea cycle; Arginine and Proline Metabolism	arginine	202	232	1.4	0.0382	0.3411
	urea	GC/MS	1176;16150869	0.97	0.6324	0.6505
	ornithine	GC/MS	6262	1.3	0.1854	0.5058
	proline	202	145742;6971047	2.38	0.0001	0.0178
	argininosuccinate	202	16950;828	0.98	0.5745	0.6403
	homocitrulline	202	65072;6991977	0.91	0.5935	0.6447
	dimethylarginine (SDMA + ADMA)	202	123831	1.14	0.5327	0.6359
	N-acetylarginine	203	67427;1615663	0.93	0.3787	0.6117
	N-acetylproline	202	322640	0.96	0.4667	0.635
	N-delta-acetylornithine*	202	9920500	1.13	0.4541	0.635
	N2,N5-diacetylornithine	202	10398396	0.92	0.1093	0.4348
	N-methyl proline	202	557	1.06	0.6623	0.6549
	3-hydroxyproline	GC/MS	559314	1.25	0.9198	0.7207
	pro-hydroxy-pro	202	11673055	1.17	0.2674	0.5528
	creatine	202	586	0.33	0.2386	0.5494

Sub Pathway	Biochemical Name	Platform	PUBCHEM	Fold of Change BCa / All CTRLs	Statistics BCA / All Ctrls p-value	q-value
Creatine Metabolism	creatinine	202	588	1.1	0.1594	0.4778
	N-carbamoylsarcosine	202	439375	1.04	0.7604	0.6782
	guanidinoacetate	202	763;3946848;4546993	0.66	0.0058	0.152
Polyamine Metabolism	guanidine	GC/MS	3520	1.14	0.3279	0.5788
	acisoga	202	129397	1.21	0.0603	0.3919
	spermine	202	1103	4.63	0.0087	0.1921
	spermidine	202	1102	1.85	0.0422	0.3474
	5-methylthioadenosine (MTA)	202	439176	0.96	0.2826	0.5528
	N-acetylputrescine	202	122356	1.03	0.7604	0.6782
	4-acetamidobutanoate	202	18189	1.12	0.0437	0.3474
Guanidino and Acetamido Metabolism	4-guanidinobutanoate	202	500	0.96	0.7393	0.6693
	guanidinosuccinate	202	97856	1.31	0.1436	0.4592
Glutathione Metabolism	5-oxoproline	203	7405	1.05	0.292	0.5579
Gamma-glutamyl Amino Acid	gamma-glutamylisoleucine*	202		1.06	0.6927	0.6583
	gamma-glutamylleucine	202	151023	1.28	0.5697	0.6377
	gamma-glutamylphenylalanine	202	111299	1.23	0.3284	0.5788
	gamma-glutamylthreonine*	202		0.99	0.5464	0.6359
	gamma-glutamyltyrosine	202	94340	1.07	0.8244	0.6964
	gamma-glutamylvaline	202		1.08	0.6673	0.6549
Dipeptide Derivative	carnosine	203	439224	1.18	0.2414	0.5494
	N-acetylcarnosine	202	9903482	0.97	0.6031	0.6465
	anserine	203	112072	0.96	0.5101	0.635
Dipeptide	alanylalanine	GC/MS	5484352	1.09	0.9502	0.7236
	alanylisoleucine	202	417358;5246008	6.54	0.0081	0.1921
	alanylleucine	202	259583	18.56	0.0004	0.0351
	alanylphenylalanine	202	2080	7.33	0.0066	0.1684
	alanylvaline	202	137276	7.13	0.0002	0.0286
	arginylleucine	202	333445	18.9	0.0087	0.1921
	arginylphenylalanine	202	4441256	176.65	0.0003	0.0286
	aspartylglycine	GC/MS	302429;5247119	1.06	0.8651	0.7059
	cyclo(gly-pro)	202	126154	1.39	0.2556	0.5528
	cyclo(leu-gly)	202	927723	0.87	0.8952	0.7163
	cyclo(leu-pro)	202	7074739	0.93	0.1798	0.502
	cyclo(L-phe-D-pro)*	202	6992198	1.43	0.9229	0.7211
	cyclo(L-phe-L-pro)	202	9837725;443440	0.79	0.4965	0.635
	glycylglycine	GC/MS	1548897;11163	1.31	0.0531	0.3801
	glycylproline	202	3013625;6993386	1.08	0.9282	0.7219
	isoleucylleucine	202	11644431	3.86	0.0011	0.0594
	isoleucylphenylalanine	202	435728	4.12	0.0014	0.0686
	leucylisoleucine	202		5.54	0.0034	0.1034
	leucylleucine	202	76807;6992072	11.16	0.0352	0.3267
	leucylphenylalanine	202	259325	4.51	0.01	0.1971
	methionylphenylalanine	202	100048	22.74	0.0508	0.3749
	phenylacetylphenylalanine	203	47583	1.2	0.3415	0.5891
	phenylalanylglutamate	203	4422358	1		
	prolylglycine	202	7408076;6426709	1.12	0.5057	0.635
	pyroglutamylglutamine	203		1.11	0.2984	0.5613
	pyroglutamylvaline	203	152416	0.94	0.3231	0.5785
	serylleucine	202		5.45	0.0017	0.0739
	serylphenyalanine	202		8.8	0.0024	0.0846
	threonylleucine	202	4420322	11.93	0.0007	0.0464
	threonylphenylalanine	202	4099799;4099798	18.64	0.0096	0.1971
	tyrosylarginine	202	2932	62.86	4.93E-07	0.0004
	valylisoleucine	202	5246012;5246013	6.53	0.0005	0.0394

Sub Pathway	Biochemical Name	Platform	PUBCHEM	Fold of Change	Statistics	
				BCa All CTRLs	BCA All Ctrls	
					p-value	q-value
	valylleucine	202	352039	16.66	0.02	0.2452
Polypeptide	Ac-Ser-Asp-Lys-Pro-OH	202	4409396	0.59	0.2276	0.5494
	asp-ser-val	202		0.7	0.3114	0.5726
Glycolysis, Gluconeogenesis, and Pyruvate Metabolism	1,5-anhydroglucitol (1,5-AG)	203		1.28	0.0932	0.4348
	glucose	GC/MS	79025	0.71	0.3049	0.567
	3-phosphoglycerate	GC/MS		2.01	0.0246	0.2641
	pyruvate	GC/MS	107735	0.89	0.5983	0.6448
	lactate	GC/MS	612	2.05	0.0016	0.0739
Pentose Metabolism	ribulose	GC/MS	79021	1.03	1	0.7367
	ribitol	GC/MS		1	0.7869	0.686
	ribonate	GC/MS	5460677	1.18	0.5304	0.6359
	xylulose	GC/MS	5289590	1.14	0.2857	0.5528
	xylonate	GC/MS	6602431	1.08	0.3249	0.5788
	xylose	GC/MS	95259	0.68	0.8461	0.703
	xylitol	GC/MS	6912	1.04	0.1272	0.4441
	arabinose	GC/MS	66308	1.08	0.4016	0.6193
	threitol	GC/MS	169019	1.1	0.2826	0.5528
	arabitol	GC/MS	94154	1.19	0.1878	0.507
	lyxose	GC/MS	65550	1.22	0.6775	0.6563
	fucose	GC/MS	3034656	1.25	0.2585	0.5528
Disaccharides and Oligosaccharides	lactose	GC/MS	6134	0.93	0.6573	0.6549
	3-sialyllactose	203	123914	1.07	0.8191	0.6962
	sucrose	203	5988	1.39	0.2498	0.5528
	glucarate 1,4-lactone	GC/MS	16219942	1.29	0.4427	0.635
Fructose, Mannose and Galactose Metabolism	fructose	GC/MS	5984	0.94	0.703	0.6599
	sorbose	GC/MS	441484	2.29	0.7551	0.6757
	sorbitol	GC/MS	107428	1.14	0.7736	0.6825
	mannose	GC/MS	161658	1	0.8244	0.6964
	mannitol	GC/MS	6251	3.17	0.5887	0.6438
	galactose	GC/MS	3037556	1.88	0.4795	0.635
	galactitol (dulcitol)	GC/MS	11850	1.65	0.0494	0.3749
	2-ketogulonate	GC/MS	102424	6.24	0.2145	0.5317
	galactonate	GC/MS	128869	1.3	0.6422	0.6524
Aminosugar Metabolism	glucosamine	GC/MS	441477	2.16	0.5102	0.635
	glucuronate	GC/MS	65041	1.56	0.0031	0.1
	N-acetylneuraminate	203		1.25	0.0683	0.4197
	6-sialyl-N-acetyllactosamine	203	16212424	1.17	0.0869	0.4348
	erythronate*	GC/MS	2781043	1.18	0.1553	0.4778
TCA Cycle	citrate	GC/MS	311	1.02	0.9392	0.7227
	cis-aconitate	203	643757	0.99	0.9945	0.7346
	isocitrate	GC/MS	1198	1.04	0.3458	0.594
	alpha-ketoglutarate	GC/MS	51	1.14	0.7709	0.6809
	succinylcarnitine	202		1.04	0.8897	0.7158
	succinate	GC/MS	1110	0.95	0.0531	0.3801
	fumarate	GC/MS		1.23	0.0715	0.424
	malate	GC/MS	525	1.3	0.5721	0.6394
	itaconate (methylenesuccinate)	GC/MS	811	0.72	0.6927	0.6583
	2-methylcitrate	GC/MS	5460420	1.11	0.2614	0.5528
	mesaconate (methylfumarate)	GC/MS		1.24	0.7446	0.6707
Oxidative Phosphorylation	phosphate	203	1061	1.25	0.3602	0.5985
Polyunsaturated Fatty Acid (n3 and n6)	arachidonate (20:4n6)	203	444899	3.45	4.17E-06	0.0017
	2-hydroxyglutarate	GC/MS	43	1.08	0.6275	0.6495
	adipate	GC/MS	196	7.58	0.1055	0.4348
	2-hydroxyadipate	GC/MS	193530	1.12	0.7656	0.6797

Sub Pathway	Biochemical Name	Platform	PUBCHEM	Fold of Change	Statistics	
				BCa / All CTRLs	BCA All Ctrls	
					p-value	q-value
Fatty Acid, Dicarboxylate	3-methyladipate	GC/MS	12292	1.26	0.4795	0.635
	pimelate (heptanedioate)	GC/MS	385	1.53	0.295	0.5579
	suberate (octanedioate)	203	10457	1.25	0.3602	0.5985
	4-octenedioate	GC/MS	11805205	1.38	0.0894	0.4348
	azelate (nonanedioate)	203	2266	0.7	0.4582	0.635
	sebacate (decanedioate)	203	5192	0.93	0.7856	0.686
	dodecanedioate	203	12736	0.87	0.4015	0.6193
	3-carboxy-4-methyl-5-propyl-2-furanpropanoate (CMPF)	203	123979	0.41	0.1116	0.4348
Fatty Acid, Amino	2-aminooctanoate	202	69522	0.97	0.5146	0.635
Fatty Acid Synthesis	malonylcarnitine	202		1.06	0.4499	0.635
	2-methylmalonyl carnitine	202		1.03	0.6623	0.6549
Fatty Acid Metabolism (also BCAA Metabolism)	butyrylcarnitine	202	439829	1.55	0.0627	0.398
	propionylcarnitine	202	107738	1.22	0.3529	0.5962
Fatty Acid Metabolism(Acyl Glycine)	hexanoylglycine	202	99463	1.09	0.5959	0.6448
Fatty Acid Metabolism(Acyl Carnitine)	acetylcarnitine	202	1	1.4	0.0566	0.3801
	hydroxybutyrylcarnitine*	202		3.19	0.1248	0.4441
	hexanoylcarnitine	202	6426853	1.27	0.0881	0.4348
	decanoylcarnitine	202	10245190	1.15	0.2704	0.5528
	laurylcarnitine	202	10427569	1.23	0.4038	0.6193
Carnitine Metabolism	deoxycarnitine	202	134	1.51	0.1765	0.4961
	carnitine	202	10917	1.33	0.2414	0.5494
	3-dehydrocarnitine*	202	6991982	1.05	0.6876	0.6566
Ketone Bodies	acetate*	203	176	1.04	0.6825	0.6563
	3-hydroxybutyrate (BHBA)	GC/MS	441	3.82	0.1921	0.5128
Fatty Acid, Monohydroxy	3-hydroxypropanoate	GC/MS	5459847	0.91	0.4253	0.625
	3-hydroxysebacate	203	3017884	1.6	0.2021	0.5244
	5-hydroxyhexanoate	203	170748	0.77	0.3494	0.5962
Inositol Metabolism	myo-inositol	GC/MS		1.8	0.1238	0.4441
	chiro-inositol	GC/MS		5.2	0.0194	0.2452
	pinitol	GC/MS		1	0.8379	0.702
	scyllo-inositol	GC/MS		1.21	0.2952	0.5579
Phospholipid Metabolism	choline	202	305	1.11	0.2527	0.5528
	choline phosphate	202	1014	1.88	0.0188	0.2452
	glycerophosphorylcholine (GPC)	202	657272	1.24	0.1533	0.4778
	ethanolamine	GC/MS		1.11	0.9337	0.7219
	phosphoethanolamine	GC/MS	5232324;1015	1.35	0.2288	0.5494
	trimethylamine N-oxide	202	1145	0.99	0.3675	0.6032
Glycerolipid Metabolism	glycerol	GC/MS	753	3.27	0.3682	0.6032
	glycerol 3-phosphate (G3P)	GC/MS	754	1.28	0.3565	0.5985
Sphingolipid Metabolism	palmitoyl sphingomyelin	GC/MS	9939941	4.71	1.27E-05	0.0034
Mevalonate Metabolism	3-hydroxy-3-methylglutarate	GC/MS	5459993	1.36	0.4667	0.635
Steroid	21-hydroxypregnenolone disulfate	203	134595	1.03	0.6775	0.6563
	5alpha-pregnan-3beta,20alpha-diol disulfate	203		1.33	0.3564	0.5985
	pregnen-diol disulfate*	203		1.29	0.2857	0.5528
	pregn steroid monosulfate*	203		1.63	0.0034	0.1034
	pregnanediol-3-glucuronide	203	123796	1.28	0.4253	0.625
	cortisol	202	5754	1.25	0.0934	0.4348
	corticosterone	202	5753	0.99	0.9117	0.7185
	cortisone	202	222786	1.12	0.5557	0.6359
	cortisone monosulfate	203	193322	1.26	0.4873	0.635
	tetrahydrocortisone	203		1.39	0.008	0.1921
	dehydroisoandrosterone sulfate (DHEA-S)	203	12594	0.86	0.5102	0.635
	epiandrosterone sulfate	203		0.95	0.7004	0.6599
	androsterone sulfate	203	159663	0.89	0.9007	0.7163

Sub Pathway	Biochemical Name	Platform	PUBCHEM	Fold of Change	Statistics	
				BCa / All CTRLs	BCA All Ctrls p-value	q-value
	4-androsten-3beta,17beta-diol disulfate 1*	203		0.91	0.7159	0.6641
	4-androsten-3beta,17beta-diol disulfate 2*	203		0.79	0.7551	0.6757
	5alpha-androstan-3beta,17alpha-diol disulfate	203		0.72	0.9637	0.7267
	5alpha-androstan-3beta,17beta-diol disulfate	203		1.13	0.6104	0.6492
	andro steroid monosulfate 1*	203		1.13	0.9172	0.7194
	andro steroid monosulfate 2*	203		1.02	0.4134	0.6197
Primary Bile Acid Metabolism	cholate	203	221493	2.25	0.2733	0.5528
	glycocholate	203	10140	2.38	0.0575	0.3803
Secondary Bile Acid Metabolism	glycolithocholate sulfate*	203	72222	0.87	0.9007	0.7163
	taurolithocholate 3-sulfate	203	440071	0.83	0.4499	0.635
	glycoursodeoxycholate	203		1.82	0.1147	0.4397
	glycohyocholate	203		1.31	0.2655	0.5528
	taurohyodeoxycholic acid	203	119046	0.53	0.3325	0.581
	glycohyodeoxycholic acid	203	114611	0.59	0.2035	0.5244
	12-dehydrocholate	203	94235	0.86	0.1078	0.4348
	glycocholenate sulfate*	203		1.04	0.5464	0.6359
	taurocholenate sulfate*	203		0.93	0.6623	0.6549
	7-ketodeoxycholate	203	188292	2.08	0.9382	0.7227
Purine Metabolism, (Hypo)Xanthine/Inosine containing	inosine	203		1.12	0.4133	0.6197
	hypoxanthine	GC/MS	790	1.1	0.5511	0.6359
	xanthine	202	1188	1.04	0.8244	0.6964
	xanthosine	202	64959	1.16	0.3977	0.6193
	urate	203		0.98	0.953	0.7251
	allantoin	GC/MS	204	0.85	0.3115	0.5726
	9-methyluric acid	203	108714	1.35	0.1613	0.4787
Purine Metabolism, Adenine containing	adenosine 3',5'-cyclic monophosphate (cAMP)	202	6076	0.95	0.9337	0.7219
	adenosine	202	60961	0.75	0.0119	0.2007
	adenine	202	190	1.37	0.0459	0.3585
	1-methyladenine	202	78821	0.99	0.9062	0.7163
	N1-methyladenosine	202	5460178	1	0.7341	0.6687
	N6-methyladenosine	202	102175	1.09	0.4292	0.6295
	N6-carbamoylthreonyladenosine	202		1.14	0.1063	0.4348
Purine Metabolism, Guanine containing	guanosine-3',5'-cyclic monophosphate (cGMP)	203	295	0.99	0.8352	0.7011
	guanosine	202	6802	1.51	0.0309	0.2986
	guanine	202	764	0.74	0.1604	0.4787
	7-methylguanine	202	11361	0.99	0.6876	0.6566
	N1-methylguanosine	202	96373	0.94	0.4334	0.63
	N2,N2-dimethylguanosine	202	92919	1.06	0.5373	0.6359
	N2,N2-dimethylguanine	202	74047	1.44	0.3215	0.578
Pyrimidine Metabolism, Orotate containing	orotate	203	967	1.39	0.4752	0.635
	orotidine	203	92751	1.6	0.7551	0.6757
Pyrimidine Metabolism, Uracil containing	uridine	203	6029	1.54	0.0024	0.0846
	uracil	GC/MS	1174	0.98	0.6275	0.6495
	pseudouridine	203		1.11	0.2585	0.5528
	5,6-dihydrouracil	202	649	1.1	0.247	0.5528
	4-ureidobutyrate	202	1571307	0.88	0.4173	0.6233
	3-ureidopropionate	202	111	1.03	0.9558	0.7251
	beta-alanine	GC/MS	239	1.45	0.1379	0.4502
	N-acetyl-beta-alanine	202	76406	0.87	0.1614	0.4787
Pyrimidine Metabolism, Cytidine containing	cytidine	202	6175	1	0.9062	0.7163
	cytosine	GC/MS	597	1.4	0.0254	0.265
	3-methylcytidine	202	10988995	1.18	0.129	0.4441
	N4-acetylcytidine	202	107461	1.22	0.015	0.2134
	thymidine	203	5789	1.35	0.1403	0.4542

Sub Pathway	Biochemical Name	Platform	PUBCHEM	Fold of Change	Statistics	
				BCa / All CTRLs	BCA All Ctrls	
					p-value	q-value
Pyrimidine Metabolism, Thymine containing	thymine	GC/MS	1135	1.14	0.1573	0.4778
	5,6-dihydrothymine	202	93556	1.11	0.8788	0.7105
	3-aminoisobutyrate	GC/MS	64956	1.93	0.2644	0.5528
Nicotinate and Nicotinamide Metabolism	quinolinate	202	1066	**1.34**	0.015	0.2134
	nicotinate	GC/MS	938	1.26	0.6775	0.6563
	nicotinate ribonucleoside*	202	161233	1.47	0.4201	0.624
	nicotinamide	202	936	1.13	0.2556	0.5528
	1-methylnicotinamide	202	10129985	0.78	0.1109	0.4348
	6-hydroxynicotinate	GC/MS	72924	1.11	0.1833	0.505
	trigonelline (N'-methylnicotinate)	202	5570	1.09	0.9007	0.7163
	nicotinurate	202	68499	0.82	0.1877	0.507
	N1-Methyl-2-pyridone-5-carboxamide	202	69698	1.06	0.9337	0.7219
Riboflavin Metabolism	riboflavin (Vitamin B2)	202	493570	0.61	0.5214	0.635
Pantothenate and CoA Metabolism	pantothenate	202	6613	0.95	0.8679	0.7059
Ascorbate and Aldarate Metabolism	gulono-1,4-lactone	GC/MS	608	**1.39**	0.0222	0.2572
	glucarate (saccharate)	GC/MS	33037	**1.57**	0.0147	0.2134
	ascorbate (Vitamin C)	GC/MS		0.73	0.4991	0.635
	threonate	GC/MS	151152	**1.33**	0.0086	0.1921
	arabonate	GC/MS	122045	1.11	0.4925	0.635
Tocopherol Metabolism	gamma-CEHC	203	133098	0.84	0.4496	0.635
	gamma-CEHC glucuronide*	203		0.95	0.6724	0.6563
	alpha-CEHC glucuronide*	203		**1.27**	0.0869	0.4348
Tetrahydrobiopterin Metabolism	biopterin	202	445040	0.92	0.7709	0.6809
	dihydrobiopterin	202	1879	0.84	0.2734	0.5528
Pterin Metabolism	pterin	202	73000	0.96	0.2826	0.5528
	neopterin	202	440842	**1.27**	0.043	0.3474
	7,8-dihydroneopterin	GC/MS	65074	1.33	0.1811	0.5022
Hemoglobin and Porphyrin Metabolism	5-aminolevulinate	GC/MS	137;7048523	1.21	0.5815	0.6421
	heme	202		**41.18**	0.0003	0.0286
	L-urobilin	202	5280818	3.03	0.7305	0.6684
Thiamine Metabolism	thiamin (Vitamin B1)	202		0.73	0.2629	0.5528
Vitamin B6 Metabolism	pyridoxine (Vitamin B6)	202	1054	1		
	pyridoxate	202	6723	0.79	0.7289	0.6677
Benzoate Metabolism	hippurate	203	464	**1.83**	0.0238	0.2617
	2-hydroxyhippurate (salicylurate)	203	10253	1.33	0.3494	0.5962
	3-hydroxyhippurate	203	450268	1.37	0.4134	0.6197
	4-hydroxyhippurate	203	151012	1.33	0.3318	0.581
	mandelate	GC/MS	1292	1.11	0.1533	0.4778
	3-hydroxymandelate	GC/MS	86957	6.74	0.4211	0.6244
	4-hydroxymandelate	GC/MS	328	1.01	0.5935	0.6447
	benzoate	GC/MS	243	**0.94**	0.0622	0.398
	benzoate 4-sulfate	203		1	0.4774	0.635
	4-hydroxybenzoate	GC/MS	135;3702506	1.36	0.8191	0.6962
	3-hydroxybenzoate	GC/MS	7420	1.05	1	0.7367
	2,4,6-trihydroxybenzoate	203	66520	0.92	0.7868	0.686
	2,3-dihydroxybenzoate	GC/MS	19	1.34	0.6347	0.652
	catechol sulfate	203	3083879	1.17	0.4094	0.6195
	O-methylcatechol sulfate	203	22473	1.2	0.2046	0.5244
	3-methyl catechol sulfate 1	203		1.12	0.6177	0.6494
	3-methyl catechol sulfate 2	203		1	0.8787	0.7105
	4-methylcatechol sulfate	203		1.12	0.1325	0.4473
	2-ethylphenylsulfate	203		2.18	0.2321	0.5494
	3-ethylphenylsulfate*	203		1.42	0.3511	0.5962
	4-ethylphenylsulfate	203		0.85	0.4499	0.635

Sub Pathway	Biochemical Name	Platform	PUBCHEM	Fold of Change BCa / All CTRLs	Statistics BCA All Ctrls p-value	Statistics BCA All Ctrls q-value
	4-vinylphenol sulfate	203	6426766	1.13	0.5887	0.6438
	4-hydroxy catechol	GC/MS	10787	1.25	0.3148	0.5749
Xanthine Metabolism	caffeine	202	2519	1.11	0.9779	0.729
	paraxanthine	202	4687	1.23	0.6775	0.6563
	theobromine	202	5429	1.26	0.7341	0.6687
	theophylline	203	2153	1.44	0.373	0.605
	1-methylurate	202	69726	1.45	0.1878	0.507
	7-methylurate	202	69160	1.33	0.1594	0.4778
	1,3-dimethylurate	203	70346	1.42	0.1973	0.5221
	1,7-dimethylurate	203	91611	**1.53**	0.0894	0.4348
	3,7-dimethylurate	203	83126	0.91	0.6423	0.6524
	1,3,7-trimethylurate	203	79437	1.05	0.9062	0.7163
	1-methylxanthine	202	80220	1.35	0.3824	0.6141
	3-methylxanthine	202	70639	1.06	0.6851	0.6566
	7-methylxanthine	202	68374	1.04	0.9862	0.7331
	5-acetylamino-6-amino-3-methyluracil	202	88299	**1.7**	0.014	0.2134
	5-acetylamino-6-formylamino-3-methyluracil	202	108214	**1.41**	0.0921	0.4348
Tobacco Metabolite	cotinine	202	854019	4.16	0.3943	0.6193
	hydroxycotinine	202	10219774	1.57	0.6401	0.6524
	cotinine N-oxide	202	9815514	2.38	0.37	0.6032
	3-hydroxycotinine glucuronide	203	183115	2.07	0.4618	0.635
Food Component/Plant	2-piperidinone	202	12665	1.7	0.1307	0.4464
	sucralose	203		0.98	0.1036	0.4348
	genistein	202	5280961	0.32	0.3265	0.5788
	Isobar: 1-kestose, levan	203		0.66	0.2279	0.5494
	2,3-butanediol	GC/MS		0.8	0.2888	0.5549
	vanillate	GC/MS	8468	0.74	0.9613	0.7259
	ethyl vanillate	GC/MS	12038	0.76	0.419	0.6236
	1,6-anhydroglucose	GC/MS	11412545	1.02	0.3215	0.578
	2,3-dihydroxyisovalerate	GC/MS	677	0.97	0.9034	0.7163
	2-isopropylmalate	203	77	0.89	0.857	0.7043
	2-oxindole-3-acetate	202	3080590	1.05	0.6825	0.6563
	3,5-dihydroxybenzoic acid	GC/MS	7424	0.59	0.2044	0.5244
	3-hydroxyindolin-2-one	GC/MS	6097	0.95	0.7237	0.6652
	4-hydroxyisoleucine	202	2773624	**0.58**	0.0191	0.2452
	4-hydroxyproline betaine	202	164642;1550239	**1.72**	0.0254	0.265
	gluconate	GC/MS	10690	**5.25**	0.0908	0.4348
	abscisate	203	5280896	0.88	0.509	0.635
	alliin	202	87310	**0.63**	0.0902	0.4348
	caffeate	GC/MS	689043	0.51	0.9057	0.7163
	chlorogenate	203	5315832	0.7	0.1462	0.4659
	ciliatine (2-aminoethylphosphonate)	GC/MS	339	0.87	0.47	0.635
	cinnamoylglycine	203	709625	0.9	0.6031	0.6465
	citraconate	GC/MS	643798	1.29	0.6523	0.6543
	cryptochlorogenic acid	203	5315599	0.59	0.1289	0.4441
	daidzein	203	5281708	0.12	0.5179	0.635
	dihydroferulic acid	203	14340	1.05	0.608	0.6475
	enterolactone	203	10685477	1.31	0.6943	0.6583
	equol glucuronide	203		0.05	0.9454	0.7236
	equol sulfate	203		0.31	0.159	0.4778
	erythritol	GC/MS		0.54	0.5839	0.6421
	fucitol	GC/MS	3429	**1.44**	0.0405	0.3474
	glucoheptose	GC/MS		**1.89**	0.0504	0.3749
	hesperetin	203	72281	1		

Sub Pathway	Biochemical Name	Platform	PUBCHEM	Fold of Change BCa All CTRLs	Statistics BCA All Ctrls p-value	q-value
	homocitrate	GC/MS	439459	1.17	0.2046	0.5244
	homostachydrine*	202	441447	1.13	0.4838	0.635
	indoleacrylate	203	5375048	1.15	0.9668	0.7267
	Isobar: dihydrocaffeate, 3,4-dihydroxycinnamate	GC/MS		0.98	0.4618	0.635
	malitol	GC/MS	3871	1.45	0.0934	0.4348
	methyl indole-3-acetate	202	74706	0.9	0.944	0.7236
	N-(2-furoyl)glycine	203	21863	1.14	0.8137	0.6938
	naringenin	203	932	0.66	0.0105	0.1971
	pyromucic acid	GC/MS	6919	1.03	0.8632	0.7056
	quinate	GC/MS		1.79	0.8137	0.6938
	resveratrol	202	445154	0.99	0.5586	0.6359
	saccharin	203	5143	5.75	0.2413	0.5494
	stachydrine	202	115244	1.49	0.0415	0.3474
	sulforaphane	202	5350	0.93	0.627	0.6495
	tartarate	GC/MS	875	1.21	0.1255	0.4441
	thymol sulfate	203		1.05	0.7405	0.6693
	trihydroxybutane	GC/MS	20497	1.03	0.3958	0.6193
Drug	Gentamycin*	202	72395	1		
	2-hydroxyacetaminophen sulfate*	203		2.64	0.0045	0.13
	2-methoxyacetaminophen sulfate*	203		4.56	0.0113	0.1971
	3-(cystein-S-yl)acetaminophen*	202	5233914	3.65	0.0006	0.0436
	3-(N-acetyl-L-cystein-S-yl) acetaminophen*	202	83967	4.39	0.0005	0.0394
	4-acetaminophen sulfate	203	83939	3.14	0.0058	0.152
	4-acetamidophenol	202	1983	3.6	0.014	0.2134
	p-acetamidophenylglucuronide	203	4022661	4.06	0.0111	0.1971
	salicyluric glucuronide*	203		2.51	0.9641	0.7267
	ibuprofen acyl glucuronide	202	163959	0.04	0.8042	0.6901
	ibuprofen	203	3672	0.98	0.5586	0.6359
	2-hydroxyibuprofen	202		0.19	0.6661	0.6549
	carboxyibuprofen	202	10444113	0.63	0.6411	0.6524
	3-hydroxyquinine	202	441264;157225	1.09	0.1032	0.4348
	4-acetylphenol sulfate	203	4684006	0.8	0.4838	0.635
	6-oxopiperidine-2-carboxylic acid	202	3014237	0.88	0.2556	0.5528
	acetylsalicylate	GC/MS	2244	0.87	0.2231	0.5461
	allopurinol	GC/MS	2094	1.1	0.7073	0.6599
	allopurinol riboside	203		1.4	0.473	0.635
	amitriptyline	202	2160	1.1	0.1032	0.4348
	asmol	202	2083	0.74	0.5527	0.6359
	atenolol	202	2249	1	0.7888	0.6862
	benzoylecgonine	202	442997	1.11	0.1032	0.4348
	desmethylnaproxen sulfate*	203	184679	6.17	0.6324	0.6505
	desvenlafaxine	202		1		
	diltiazem	202	39186	0.9	0.3976	0.6193
	diphenhydramine	202	3100	0.92	0.6195	0.6494
	doxycycline	202		0.12	0.7175	0.6642
	doxylamine	202	3162	1		
	erlotinib	203	176870	1		
	escitalopram	202	146570	1.17	0.7204	0.6651
	fexofenadine	202	3348	0.97	0.5586	0.6359
	fluoxetine	202	3386	1.29	0.0198	0.2452
	furosemide	203	3440	0.21	0.8389	0.702
	gabapentin	203	3446;6919078	3	0.2752	0.5528
	Gemfibrozil	203	3463	0.99	0.5586	0.6359
	hydrochlorothiazide	203	3639	0.17	0.2146	0.5317

Sub Pathway	Biochemical Name	Platform	PUBCHEM	Fold of Change BCa / All CTRLs	Statistics BCA All Ctrls p-value	q-value
	hydroquinone sulfate	203	161220	1.07	0.4499	0.635
	hydroxyurea	GC/MS	3657	1		
	imipramine	202	3696	1		
	isosorbide	GC/MS	12597	1.05	0.8966	0.7163
	ketamine	202	3821	1		
	lidocaine	202	3676	38.75	0.0106	0.1971
	lipitor	203	60823	0.96	0.6644	0.6549
	meprobamate*	202	4064	1		
	metformin	202	4091	0.03	0.2396	0.5494
	methamphetamine	202	1206	1		
	metoprolol	202	4171	0	0.1335	0.4473
	metoprolol acid metabolite*	202	62936	0.15	0.1432	0.4592
	mirtazapine	202	4205	0.94	0.5586	0.6359
	naproxen	GC/MS	156391	1.19	0.7888	0.6862
	N-desmethyl rosuvastatin	203	9956224	1	0.4748	0.635
	N-ethylglycinexylidide*	202	24415	2.81	0.0335	0.32
	nicotine	202	89594	0.93	0.2374	0.5494
	norfluoxetine	202	4541	1.12	0.0198	0.2452
	ofloxacin	202	3288597;4583	1		
	oxypurinol	202	4644	1	0.7069	0.6599
	paroxetine	202	43815	1	0.5586	0.6359
	phenobarbital	203	4763	1		
	pivaloylcarnitine	202	126894	1.46	0.1093	0.4348
	pseudoephedrine	202	7028	0.99	0.5586	0.6359
	quinine	GC/MS	2728270	1.01	0.1032	0.4348
	ranitidine	202	3001055	0.98	0.5586	0.6359
	salicylate	GC/MS	338	2.38	0.8843	0.7128
	sertraline	202	68617	1		
	triamterene	202	5546	1		
	colchicine	202	6167	1	0.5586	0.6359
	3-(N-acetyl-L-cystein-S-yl) acetominophen	203		3.82	0.0018	0.0739
Chemical	tetraethylene glycol	202	8200	0.72	0.9007	0.7163
	pentaethylene glycol	202	62551	0.55	0.6153	0.6494
	hexaethylene glycol	202	17472	0.44	0.6724	0.6563
	heptaethylene glycol	202	79718	0.36	0.6548	0.6549
	octaethylene glycol	202	78798	0.35	0.565	0.6368
	diglycerol	202	42953	0.98	0.7393	0.6693
	1,2-propanediol	GC/MS		3.91	0.0437	0.3474
	3-hydroxypyridine	GC/MS	7971	1.37	0.5983	0.6448
	O-sulfo-L-tyrosine	203		1.06	0.1699	0.4876
	2-oxo-1-pyrrolidinepropionate	202	3146688	1.35	0.5191	0.635
	ethyl glucuronide	203	152226	0.54	0.8043	0.6901
	2-aminophenol sulfate	203	181670	2.02	0.5236	0.635
	2-hydroxyisobutyrate	GC/MS	11671	1.16	0.0566	0.3801
	N2-(3-cyclopentylpropanoyl)glutamine	203		1.06	0.9558	0.7251
	S-(3-hydroxypropyl)mercapturic acid	202	3371179	1.4	0.8788	0.7105
	dimethyl sulfone	202	6213	1.04	0.5503	0.6359
	ectoine	202	126041;6993223	0.97	0.7185	0.6642
	glycerol 2-phosphate	GC/MS	2526	1.01	0.6226	0.6494
	glycolate (hydroxyacetate)	GC/MS	3698251;757	1.02	0.5839	0.6421
	metacryloyl glycine	202	152645	0.86	0.4838	0.635
	phenylcarnitine*	202		1.56	0.1656	0.4823
	succinimide	GC/MS	11439	1.24	0.0234	0.2605
	triethanolamine	GC/MS		0.8	0.5983	0.6448

Sub Pathway	Biochemical Name	Platform	PUBCHEM	Fold of Change	Statistics	
				BCa / All CTRLs	BCA / All Ctrls	
					p-value	q-value
	trizma acetate	GC/MS	81291;16218782	0.99	0.9325	0.7219
